# Variation in Broccoli Cultivar Phytochemical Content under Organic and Conventional Management Systems: Implications in Breeding for Nutrition

**DOI:** 10.1371/journal.pone.0095683

**Published:** 2014-07-16

**Authors:** Erica N. C. Renaud, Edith T. Lammerts van Bueren, James R. Myers, Maria João Paulo, Fred A. van Eeuwijk, Ning Zhu, John A. Juvik

**Affiliations:** 1 Wageningen UR Plant Breeding, Plant Sciences Group, Wageningen University, Wageningen, The Netherlands; 2 Department of Horticulture, Oregon State University, Corvallis, Oregon, United States of America; 3 Biometris, Plant Sciences Group, Wageningen University, Wageningen, The Netherlands; 4 Department of Crop Sciences, University of Illinois, Urbana, Illinois, United States of America; Cairo University, Egypt

## Abstract

Organic agriculture requires cultivars that can adapt to organic crop management systems without the use of synthetic pesticides as well as genotypes with improved nutritional value. The aim of this study encompassing 16 experiments was to compare 23 broccoli cultivars for the content of phytochemicals associated with health promotion grown under organic and conventional management in spring and fall plantings in two broccoli growing regions in the US (Oregon and Maine). The phytochemicals quantified included: glucosinolates (glucoraphanin, glucobrassicin, neoglucobrassin), tocopherols (δ-, γ-, α-tocopherol) and carotenoids (lutein, zeaxanthin, β-carotene). For glucoraphanin (17.5%) and lutein (13%), genotype was the major source of total variation; for glucobrassicin, region (36%) and the interaction of location and season (27.5%); and for neoglucobrassicin, both genotype (36.8%) and its interactions (34.4%) with season were important. For δ- and γ- tocopherols, season played the largest role in the total variation followed by location and genotype; for total carotenoids, genotype (8.41–13.03%) was the largest source of variation and its interactions with location and season. Overall, phytochemicals were not significantly influenced by management system. We observed that the cultivars with the highest concentrations of glucoraphanin had the lowest for glucobrassicin and neoglucobrassicin. The genotypes with high concentrations of glucobrassicin and neoglucobrassicin were the same cultivars and were early maturing F_1_ hybrids. Cultivars highest in tocopherols and carotenoids were open pollinated or early maturing F_1_ hybrids. We identified distinct locations and seasons where phytochemical performance was higher for each compound. Correlations among horticulture traits and phytochemicals demonstrated that glucoraphanin was negatively correlated with the carotenoids and the carotenoids were correlated with one another. Little or no association between phytochemical concentration and date of cultivar release was observed, suggesting that modern breeding has not negatively influenced the level of tested compounds. We found no significant differences among cultivars from different seed companies.

## Introduction

Organic food consumption is in part driven by consumer perception that organic foods are more nutritious and simultaneously less potentially harmful to human health [1]–[2]. Studies, such as Smith-Sprangler et al. [3], have concluded that there is little evidence for differences in health benefits between organic and conventional products, but other studies have indicated that organic vegetables and fruits contain higher concentrations of certain plant phytochemicals associated with health promotion than those produced conventionally [4]–[8]. A number of these compounds are produced by plants in response to environmental stress or pathogen infection, providing a potential explanation of why concentrations of these compounds might be higher in plants grown in organic systems without application of pesticides [9]. In addition, higher phytochemical levels may be due to the effects that different fertilization practices have on plant metabolism. Synthetic fertilizers used in conventional agriculture are more readily available to plants than organic fertilizers [10]. Nutrients derived from organic fertilizers need to be mineralized, and the availability of these nutrients depends on soil moisture, temperature and level of activity of soil organisms [11]. Conventional systems seek to maximize yields, resulting in a relative decrease of plant phytochemicals and secondary metabolites [12]–[15]. Correspondingly, compounds such as phenolics, flavonoids, and indolyl glucosinolates may be induced by biotic or abiotic stress [16]–[17].

Broccoli is an abundant source of nutrients, including provitamin A (β-carotene), vitamin C (ascorbate), and vitamin E (tocopherol) [18]. It is also a source of phytochemicals associated with health benefits and these include glucosinolates, carotenoids, tocopherols, and flavonoids [19]–[21]. Verhoeven et al. [22], Keck and Finley [23] and Here and Büchler [24], reported that diets rich in broccoli reduce cancer incidence in humans. Strong associations between consumption level and disease risk reduction exists for glucosinolates (anti-cancer), tocopherols (cardiovascular), and the carotenoids (eye-health) [25].

Sulfur containing glucosinolates are found in the tissues of many species of the *Brassicaceae* family. When glucosinolates are consumed, they are hydrolyzed into isothiocyanates (ITC) and other products that up-regulate genes associated with carcinogen detoxification and elimination. Aliphatic glucoraphanin (up to 50% of total glucosinolates) and the indolylic glucosinolates, glucobrassicin and neoglucobrassicin are abundant in broccoli florets [20], [19], . Glucoraphanin is hydrolyzed either by the endogenous plant enzyme myrosinase [27]–[28] or by gut microbes to produce sulforaphane, an ITC. The indole glucosinolates are tryptophan-derived in a similar but alternate biosynthetic pathway [29]. The health promoting effects of the indolyl glucosinolates are attributed to indole-3-carbinol, a hydrolysis product of glucobrassicin, N-methoxyindole-3-carbinol and neoascorbigen, hydrolysis products from neoglucobrassicin, and the catabolic products derived from alkyl glucosinolates. Clinical studies have shown that the glucosinolate hydrolysis products reduce the incidence of certain forms of cancer (e.g., prostate, intestinal, liver, lung, breast, bladder) [30]–[35]. The lipophilic phytonutrients found in broccoli include the carotenoids lutein, zeaxanthin, β-carotene, and tocopherols (forms of vitamin E) [36]–[37]. In addition to their role as vitamins, these compounds are powerful antioxidants [38]–[39]. Consumption of vegetables high in tocopherols and carotenoids has decreased the incidence of certain forms of cancer [40]. Lutein and zeaxanthin protect against development of cataracts and age-related macular degeneration [41]. Tocopherols have also been associated with reduced risk of cardiovascular disease by preventing oxidative modification of low-density lipoproteins in blood vessels [42].

The genetic potential for high nutrient content has long been a concern of the organic industry in order to meet the expectations of organic consumers. This has often been manifested by questioning whether modern elite cultivars may have lower levels of nutritional content than older open pollinated cultivars. Indirect evidence supporting this argument comes from Davis et al. [43], who compared USDA nutrient content data for 43 garden crops released between 1950 and 1999. Statistically significant decreases were noted for six nutrients (protein, calcium, potassium, iron, riboflavin, and ascorbic acid), with declines ranging from 6% for protein to 38% for riboflavin. Crop varieties in 1950 had been bred to be adapted to specific regions and a relatively low input agriculture system, but contemporary cultivars are selected for yield, disease resistance, broad adaptation to high input agriculture systems, and for increased ‘shipability’ and shelf life. Traka et al. [44] recommend breeding with greater genetic diversity when the goal is enhanced phytochemical content by exploiting wild crop relatives. The genotype is important in determining the level of nutrients in a crop cultivar [45]–[47]. What is unclear, however, is whether the nutritional content of a cultivar is associated with certain genotypic categorization, e.g. old versus modern, open pollinated versus F_1_ hybrid cultivars. In addition, there is no clear differentiation as to what extent nutritional content in a crop is determined by genotypic or by field management factors or by the interaction of both. Some studies comparing performance of genotypes in organic and conventional production systems have shown that for certain agronomic traits, cultivars perform differently between the two production systems (e.g. for winter wheat: Murphy et al. [48], Baresel et al. [49]; for lentils: Vlachostergios et al. [50]; for maize: Goldstein et al.[51]), while others have shown no differences in ranking performance (for maize: Lorenzana and Bernardo [52]; for onions: Osman et al. [53]; for cereals: Przystalski et al.[54]). The results of these studies have profound implications for organic cultivar selection and breeding strategies and raise questions as to the need for cultivars to be bred with broad adaptability or specific adaptation for the requirements of regional organic production and for designing breeding programs that optimize phytochemicals in an adapted management system.

Previous studies comparing organically versus conventionally grown broccoli for nutritional quality have been ‘market basket’ (off-the-shelf) studies [55]–[56]. Harker [57] explained that the limitation of market basket studies is that they either have purchased the products from the store shelf and cannot relate differences to specific growing conditions or that the number of cultivars is too small to generalize the results. While other studies have compared cultivars from one production season time period to another, knowledge of the actual cultivar and production system (soil quality, temperature, rainfall) was not available [58], [43]. The concentrations and form of health-promoting nutrients in *Brassica* vegetables have been reported to vary significantly due to (1) genotype (cultivar and genotypic class) [59], [20], [26], [60], [21], [37], [61], [44], (2) environmental conditions such as season [62]–[67], light [19], max/min temperature, irrigation [68]–[69], (3) genotype by environment interactions [19], [70]–[71]; (4) management system including soil fertility [72]–[73], organic versus conventional [13], [74]–[75], days to harvest [63]–[64], and (5) post-harvest management [76]–[77]. Identifying specific growing conditions and genotypes that produce cultivars with varying phytochemical content and putative disease-prevention activity could offer value-added commercial opportunities to the seed and food industry.

In addition to research conducted on how broccoli genotypes, management system and environment interact for horticultural traits [78], we address in this paper the question of how do genotypes, management system and environment interact to determine the nutritional contributions of broccoli to the human diet. We studied the relative importance and interaction among genotypes (cultivars, genotypic classes) and environment {management system [M: organic (O) or conventional (C)], season (S, a combination of year and season within year, i.e., fall 2006, spring 2007, fall 2007, spring 2008), location (E)} in a set of 23 broccoli cultivars for floret glucosinolate, tocopherol and carotenoid concentrations grown under organic and conventional production systems in two contrasting broccoli production regions of the US: Oregon and Maine. Specifically we addressed the following questions: (1) what is the impact of organic management system compared to the environmental factors including climatic region, season and their interactions [Genotype (G) x Environment (E) x Management System (M)]? (2) is there a significant difference in phytochemical content between different genotypes and genotypic classes (old and modern cultivars; open pollinated and F_1_ hybrid cultivars; early and late maturing cultivars; and between different commercial seed sources)? (3) what is the best selection environment for a broccoli breeding program for enhanced phytochemical content?

## Materials & Methods

### Plant Material and Field Trial Locations

Twenty-three broccoli cultivars including open pollinated (OP) cultivars, inbred lines, and F_1_ hybrids were included in field trials (**Table 1**). Cultivars were grown in a randomized complete block design with three replicates in Maine (ME)-Monmouth (Latitude 44.2386^o^N, Longitude 70.0356^o^W); and Oregon (OR)-Corvallis (Latitude 44.5647^o^N, Longitude123.2608^o^W)] with each location including organically (O) and conventionally (C) managed treatments. Plots contained 36 plants, planted in three rows of 12 plants at 46 cm equidistant spacing within and between rows. The 2006 trials had only 18 of the 23 entries, and the Oregon 2006 trial had only two replicates at the organic location. Field trials were conducted for three consecutive years with one production cycle in Fall 2006, two production cycles in Spring and Fall 2007 and one production cycle in Spring 2008. The primary management differences between the organic and conventional field trial sites are outlined in **Table S1 in File S1**, which describes the production system, soils, fertility applications, the applied supplemental irrigation, and weather conditions for the area of study. Further details of the field design are reported in Renaud et al. [78].

**Table 1:** Overview of commercially available broccoli cultivars, showing origin, main characteristics, included in paired organic - conventional field trials 2006–2008.

Cultivar	Abbreviation	Origin	Cultivar Type^a^	Date of Market Entry	Maturity Classification^b^
Arcadia	ARC	Sakata	F_1_	1985	L
B1 10	B11	Rogers	F_1_	1988	M
Batavia	BAT	Bejo	F_1_	2001	M
Beaumont	BEA	Bejo	F_1_	2003	L
Belstar	BEL	Bejo	F_1_	1997	L
Diplomat	DIP	Sakata	F_1_	2004	L
Early Green	EGR	Seeds of Change	OP	1985	E
Everest	EVE	Rogers	F_1_	1988	E
Fiesta	FIE	Bejo	F_1_	1992	L
Green Goliath	GRG	Burpee	F_1_	1981	M
Green Magic	GRM	Sakata	F_1_	2003	M
Gypsy	GYP	Sakata	F_1_	2004	M
Imperial	IMP	Sakata	F_1_	2005	L
Marathon	MAR	Sakata	F_1_	1985	L
Maximo	MAX	Sakata	F_1_	2004	L
Nutribud	NUT	Seeds of Change	OP	1990	E
OSU OP	OSU	Jim Myers, OSU	OP	2005	E
Packman	PAC	Petoseed	F_1_	1983	E
Patriot	PAT	Sakata	F_1_	1991	M
Patron	PAN	Sakata	F_1_	2000	M
Premium Crop	PRC	Takii	F_1_	1975	E
USVL 048	U48	Mark Farnham, USVL	Inbred	not released	L
USVL 093	U93	Mark Farnham, USVL	Inbred	not released	M

a Cultivar Type: F_1_: hybrid; OP: Open Pollinated; Inbred.

b Maturity Classification: E: Early; M: Mid; L: Late.

### Field Data Collection

As plots approached maturity they were evaluated three times a week for field quality and broccoli heads that had reached commercial market maturity (approximately 10 to 12 cm in diameter for most of the cultivars while retaining firmness). Field quality traits evaluated on a 1 to 9 ordinal scale included head color, bead size, and bead uniformity. Average head weight was determined by taking the mean of the five individual heads per plot. Head diameter averaged for five heads at harvest maturity from each plot. Maturity was based on days to harvest from transplanting date. Detailed procedures and horticulture trait performance data are reported in Renaud et al. [78].

### Broccoli Floret Samples and glucosinolate, tocopherol, and carotenoid analysis

In order to analyse nutritional compounds of the broccoli heads, the following procedure was followed: As plots approached maturity, five broccoli head tissue samples were harvested fresh from each subplot at each trial location and were composited into a single sample per replication. The samples were frozen at −20°C and shipped in a frozen state to the University of Illinois, Urbana-Champaign where they were freeze-dried and assessed for nutritional phytochemicals. Each sample was analyzed for the glucosinolates (glucoraphanin, glucobrassicin and neoglucobrassicin), carotenoids (β-carotene, lutein, and zeaxanthin), and tocopherols (δ-, γ-, α- tocopherol) by high-performance liquid chromatography (HPLC) analysis using analytical protocols described in Brown et al. [19] for glucosinolates, and Ibrahim and Juvik [37] for tocopherols and carotenoids. Glucosinolates in lyophilized floret tissue samples were extracted and analysed by HPLC using a reverse phase C18 column. Three hundred mg samples of broccoli floret tissue were weighed out for extraction and the HPLC quantification of the tocopherols and carotenoids.

### Statistical Analysis

Various linear mixed models were used for the analysis of trait variation. We followed the same methodology as described in Renaud et al. [78], which was comparable to the approach followed by Lorenzana and Bernardo [52]. For fitting the linear mixed models, GenStat 15 (VSNi, 2012) was used. The models followed the set-up:
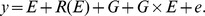



Here *y* is the phytochemical response. Term *E* represents the environment in a very general sense, it includes all main effects and interactions of Season (*S*), Location (*L*) and Management (*M*). For analyses per location, the terms involving *L* were dropped. Similarly, for analyses regarding a specific management regime, the terms involving *M* were dropped. Term *R(E)* is the effect of replicate within environment, and there were two or three replicates in individual trials. *G* and *G*×*E* are genotype and genotype by environment interaction effects, respectively. Finally *e* is a residual.

Variance components were reported as coefficients of variation, i.e., *CV*  = 100*√V*/x with *V* the variance corresponding to specific effects and x the trait mean. Repeatability was calculated from the variance components in its most general form as *H^2^ = V_G_/(V_G_ V_GL_/nL + + V_GS_/nS + V_GM_/nM + V_GLS_/(nL.nS) + V_GLM_/(nL.nM) + V_GSM_/(nS.nM) + V_GLSM_/(nL.nS.nM) + V_e_/(nL.nS.nM.nR))*, where the variance components correspond to the terms in the mixed model above. The terms *nL, nS, nM* and *nR* stand for the number of locations (2: Maine and Oregon), number of ‘seasons’ (4: Fall 2006, Spring 2007, Fall 2007, Spring 2008), management (2; organic and conventional), and replicates (2 or 3).

Genotypic means were calculated by taking genotypic main effects fixed instead of random in the mixed models above. Pairwise comparisons between genotypic means were performed using GenStat procedure *VMCOMPARISON.* Correlations on the basis of genotypic means were referred to as genetic correlations. Genotypic stabilities under organic and conventional conditions were calculated as the variance for individual genotypes across all trials in the system.

To assess the feasibility of selection for organic conditions (the target environment) under conventional conditions, we calculated the ratio of correlated response (for organic conditions using conventional conditions), CR, to direct response (for organic conditions in organic conditions), DR, as the product of the genetic correlation between organic and conventional systems (*r_G_*) and the ratio of the roots of conventional and organic repeatabilities (*H_C_* and *H_O_* respectively): *CR*/*DR*  =  *r_G_ H_G_*/*H_O_*. A ratio smaller than 1 indicates that selection is better done directly under organic conditions when the aim is indeed to improve the performance in organic conditions.

## Results

### Comparison of phytochemicals means over the environments

#### Glucosinolates

Across all trials, glucoraphanin levels were comparable between locations and seasons but were more variable at the individual location and season trial analysis level (**Table 2**). Glucoraphanin, glucobrassicin and neoglucobrassicin levels were comparable between organic and conventional treatments. Comparisons of organic versus conventional by location and season for the glucosinolate phytochemicals are presented in **Figure S1**. Comparable levels of glucosinolates were observed in the organic - conventional comparisons within locations and seasons.

**Table 2:** Trait means^1^ of phytochemicals of 23 broccoli cultivars grown across four pair combinations of location (Maine/Oregon), season (Fall/Spring) two-years combined and management system (Conventional/Organic), 2006–2008.

	Maine									Oregon								
	Fall				Spring					Fall				Spring				
	2006–2007 Combined				2007–2008 Combined				Mean	2006–2007 Combined				2007–2008 Combined				Mean
	C		O		C		O			C		O		C		O		
Glucoraphanin	5.31	^e^	3.77	^bc^	3.56	^b^	4.06	^c^	4.18	3.46	^b^	3.03	^a^	4.64	^d^	4.51	^d^	3.91
Glucobrassicin	1.06	^b^	0.90	^a^	1.45	^c^	1.33	^c^	1.19	5.14	^f^	5.51	^g^	2.24	^d^	2.70	^e^	3.90
Neoglucobrassicin	0.46	^a^	0.40	^a^	2.16	^c^	1.85	^b^	1.22	2.34	^c^	3.20	^d^	4.32	^e^	5.10	^f^	3.74
δ-Tocopherol	2.34	^c^	2.77	^d^	1.91	^b^	1.70	^a^	2.18	3.53	^e^	3.66	^e^	1.91	^b^	2.24	^c^	2.83
γ-Tocopherol	4.67	^c^	4.40	^c^	2.63	^a^	2.98	^b^	3.67	8.48	^d^	8.73	^d^	3.31	^b^	3.22	^b^	5.94
α-Tocopherol	25.83	^a^	27.33	^a^	38.61	^b^	40.51	^bc^	33.07	43.04	^c^	43.20	^c^	40.52	^bc^	42.25	^c^	42.25
Lutein	11.49	^a^	12.47	^a^	15.53	^b^	15.93	^b^	13.85	15.91	^b^	16.04	^b^	16.48	^b^	17.81	^c^	16.56
Zeaxanthin	0.81	^a^	0.83	^ab^	0.87	^ab^	0.88	^b^	0.85	0.83	^ab^	0.84	^ab^	1.02	^c^	1.02	^c^	0.93
β-Carotene	12.98	^a^	13.25	^a^	28.73	^c^	29.71	^c^	21.16	29.10	^c^	30.10	^c^	25.16	^b^	25.80	^b^	27.54

1 Values in the table are means. Means of the same letter in the same row are not significantly different at the P<0.05 level.

#### Tocopherols

Across trials compared regionally, Oregon had higher levels of all three tocopherols compared to Maine (**Table 2** **, Figure S2**). The tocopherols δ- and γ- were higher in Fall compared to Spring, but not so for α-tocopherol (**Figure S2**). Organic and conventional levels for all tocopherol concentrations were in the same range and not significantly different. When the three tocopherols were analysed by organic versus conventional within location and season, there were no clear significant differences in management system across the season and location combinations (**Table 2** **,** **Figure S2**).

#### Carotenoids

Overall, Oregon had higher levels of lutein and β-carotene compared to Maine (**Table 2** **, Figure S3**) and comparative levels of zeaxanthin (**Table 2** **, Figure S3**). Spring produced higher levels of all carotenoids compared to Fall levels in contrast to the glucosinolates and the δ- & γ- tocopherol concentrations. There were no significant differences between organic and conventional for any carotenoid measured. When carotenoids were analysed by management system within location and season, β-carotene showed significantly lower levels in Maine in the Fall compared to other location and season combinations (**Figure S3**).

### Partitioning of variance components

#### Glucosinolates

For glucoraphanin across all trials in both regions, Genotype (G) main effect accounted for the largest proportion of variance, followed by G×L×S interaction (**Table 3**). There was no Management (M) main effect, but M contributed to the three (L×S×M and G×S×M) and four-way interactions (G×L×S×M). In contrast to glucoraphanin, Location (L) had the largest effect for glucobrassicin and neoglucobrassicin across all trials in both regions, followed by the L×S interactions. For neoglucobrassicin the S and G main effect was more important than for glucobrassicin. When trials were further partitioned by location, a G and S main effect was apparent for neoglucobrassicin in both locations; for glucobrassicin the S main effects was only apparent in Oregon and not in Maine (**Table S2 in File S1**). There was M main effect for glucobrassicin and neoglucobrassicin, but not for glucoraphanin, and no G×M interaction for all glucosinolates.

**Table 3:** Partitioning of variance components (%) presented as coefficients of variation for phytochemicals of 23 broccoli cultivars grown across eight pair combinations of location (Maine/Oregon), season (Fall/Spring) and management system (Conventional/Organic), 2006–2008.

	Location (L)	Season (S)	Management (M)	L×S	L×M	S×M	L×S×M	L×S×M×R Rep (R)	Genotype (G)	G×L	G×S	G×M	G×L×S	G×L×M	G×S×M	G×L×S×M	Residual
Glucoraphanin	0.01	5.45	0.00	7.20	0.01	0.00	11.86	1.56	17.45	0.01	0.01	0.00	15.97	0.01	7.49	12.62	11.94
Glucobrassicin	36.00	0.00	0.00	27.51	5.58	4.18	1.34	1.86	9.42	7.77	0.01	0.00	13.84	0.00	0.00	10.63	10.91
Neoglucobrassicin	36.81	13.51	0.00	34.36	8.47	6.84	4.50	4.76	15.16	6.24	0.01	0.00	16.40	0.00	0.01	13.40	15.80
δ-Tocopherol	6.83	35.22	0.43	7.87	0.01	0.01	3.39	0.01	5.57	5.65	6.01	0.00	13.65	0.01	0.00	12.21	12.74
γ-Tocopherol	12.02	19.09	0.01	12.11	0.01	3.64	4.47	2.12	13.79	4.85	15.82	0.00	12.95	0.00	0.01	11.08	12.03
α-Tocopherol	6.73	0.01	0.28	10.20	0.01	0.01	0.01	1.29	2.79	3.42	0.01	0.97	10.07	0.00	0.00	8.18	8.92
Lutein	3.71	4.91	0.00	7.52	0.01	2.70	1.85	1.40	13.03	4.14	0.01	0.01	10.76	0.73	0.01	9.21	9.95
Zeaxanthin	1.97	11.55	0.01	3.99	0.01	0.00	0.01	0.00	8.44	3.18	0.01	1.05	6.91	0.01	0.83	8.52	11.36
β-Carotene	4.61	0.00	0.00	17.84	0.01	0.70	0.01	0.71	8.41	4.45	0.00	2.31	11.32	4.63	0.65	12.83	10.99

#### Tocopherols

For δ- and γ-tocopherol across all trials in both regions, the Season (S) main effect accounted for the largest proportion of variance (**Table 3**). In contrast the proportion of the variation associated with S for α-tocopherol across all trials was minor. For all three tocopherols there was minor to no M effect, but a large L main effect, being the greatest for γ-tocopherol. The G main effect showed a similar pattern to L.

#### Carotenoids

For all three carotenoids across all trials in both regions, the G main effect described a significant component of total variance and was of largest influence for lutein (**Table 3**). The S main effect played an important role for zeaxanthin, and to a lesser extent for lutein but not for β-carotene. For all three carotenoids the L effect was minor, but the L×S interaction for β-carotene was relatively large and mostly associated with Maine (**Table S2 in File S1**). There was no M main effect; only for β-carotene was there a small effect of the G×M interaction (mainly driven by Maine).

### Repeatability, genetic correlation and ratio of correlated response to direct response

#### Organic versus conventional

In the present study, we were able to estimate the proportion of the genotypic variance relative to phenotypic variance, but because we did not have a genetically structured breeding population, we apply the term repeatability rather than broad sense heritability. Of the phytochemicals studied, repeatabilities for concentrations of seven of the nine were comparable or higher in organic compared to conventional systems (**Table 4**). Only for glucobrassicin and δ-tocopherol was repeatability under organic conditions lower than under conventional. In the analyses δ- and α-tocopherol had relatively low repeatabilities. The highest repeatabilities were for glucoraphanin (0.82–0.84), neoglucobrassicin (0.75–0.76), γ-tocopherol (0.72–0.75), lutein (0.83–0.85) and zeaxanthin (0.76–0.77). Genetic correlations were high between organic and conventional for the glucosinolates, γ-tocopherol and lutein (0.84–0.95), while δ-tocopherol, α-tocopherol, zeaxanthin and β-carotene were lower (0.63–0.77). The ratio of the correlated response to direct response for selection in the organic system was less than 1.0 for all traits.

**Table 4:** Repeatabilities, genetic correlation and ratio of correlated response to direct response for broccoli phytochemicals comparing organic versus conventional management systems over all trial season/location combinations, 2006–2008.

	Repeatability (H)			
	C	O	r_A_ a	CR_org_/R_org_ b
Glucoraphanin	0.84	0.82	0.84	0.83
Glucobrassicin	0.70	0.64	0.88	0.84
Neoglucobrassicin	0.75	0.76	0.94	0.94
δ-Tocopherol	0.50	0.42	0.73	0.66
γ-Tocopherol	0.75	0.72	0.95	0.93
α-Tocopherol	0.23	0.35	0.61	0.76
Lutein	0.83	0.85	0.93	0.94
Zeaxanthin	0.76	0.77	0.77	0.78
β-Carotene	0.62	0.72	0.63	0.68

a Average genetic correlation between conventional and organic production systems across locations.

b Ratio of correlated response to direct response.

#### By location and season

For the glucosinolates, glucoraphanin and glucobrassicin repeatability at each location, season and treatment trial were comparable and generally high (0.83–0.97) between organic and conventional trials, while no clear trend for neoglucobrassicin repeatabilities was observed between organic and conventional aside from being much lower than glucoraphanin and glucobrassicin (**Table S3 in File S1**). For γ- and α-tocopherol, repeatabilities were comparable between organic and conventional, while for δ-tocopherol repeatabilities were comparable between systems or higher in conventional except for one paired trial. For the carotenoids, repeatabilities were comparable or higher in organic for all paired trials, while for lutein in seven of the eight paired trials organic was comparable or greater than conventional. Repeatabilities for zeaxanthin concentrations were comparable for six of the eight paired trials.

### Comparison of cultivar ranking for phytochemical concentration and stability across trials

**Figure 1:**
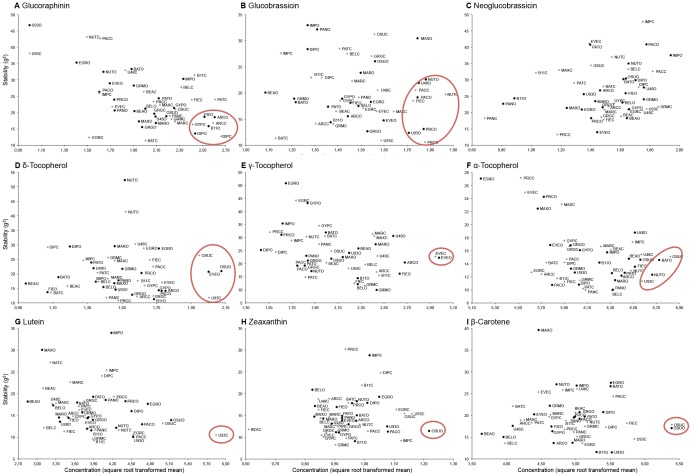
Broccoli cultivar stability from trials conducted in two locations over four seasons with two management systems plotted against phytochemical content. A. Glucoraphanin, B. Glucobrassicin, C. Neoglucobrassicin, D. δ-tocopherol, E. γ -tocopherol, F. α-tocopherol, G. Lutein, H. Zeaxanthin, I. β-carotene. See Table 1 for cultivar name abbreviations. The C or O at the end of the cultivar abbreviation indicates conventional or organic management system, respectively.

To determine trends in cultivars with both the highest concentration of phytochemical groups most stable across locations, seasons and production systems, phytochemical concentrations were plotted against stability per genotype across trials. A group of cultivars were identified as both highest in concentration and most stable and are indicated in the highlighted ‘red circle’ per phytochemical (**Figure 1A–I**). For glucoraphanin, the same group of cultivars had both the highest concentrations and were the most stable across production systems (**Figure 1A** **; Table S4 in File S1**). While for glucobrassicin, a different set of cultivars had the highest concentrations across production systems (**Figure 1B** **; Table S5 in File S1**). Overall stability of all cultivars across production system was less related to cultivar mean concentrations for glucobrassicin than for glucoraphanin. None of the cultivars with the highest concentration for neoglucobrassicin were in the top quartile for stability across trials; all cultivars with the highest neoglucobrassicin content were in the bottom half for stability (**Figure 1C** **; Table S6 in File S1**). Some but not all cultivars that had the highest concentrations of α-tocopherol were among the top group for δ- and/or γ-tocopherol. There was no relationship between δ-tocopherol concentrations and stability, but both γ- and α- tocopherols had higher concentrations associated with greater stability (**Figure 1D–F** **; Tables S7–9 in File S1**). Open pollinated and early maturing cultivars had the highest and most stable concentrations for all carotenoids. (**Figure 1G–I** **; Tables S10–12 in File S1**).

### Comparison of phytochemical concentration by genotype classification

**Figure 2:**
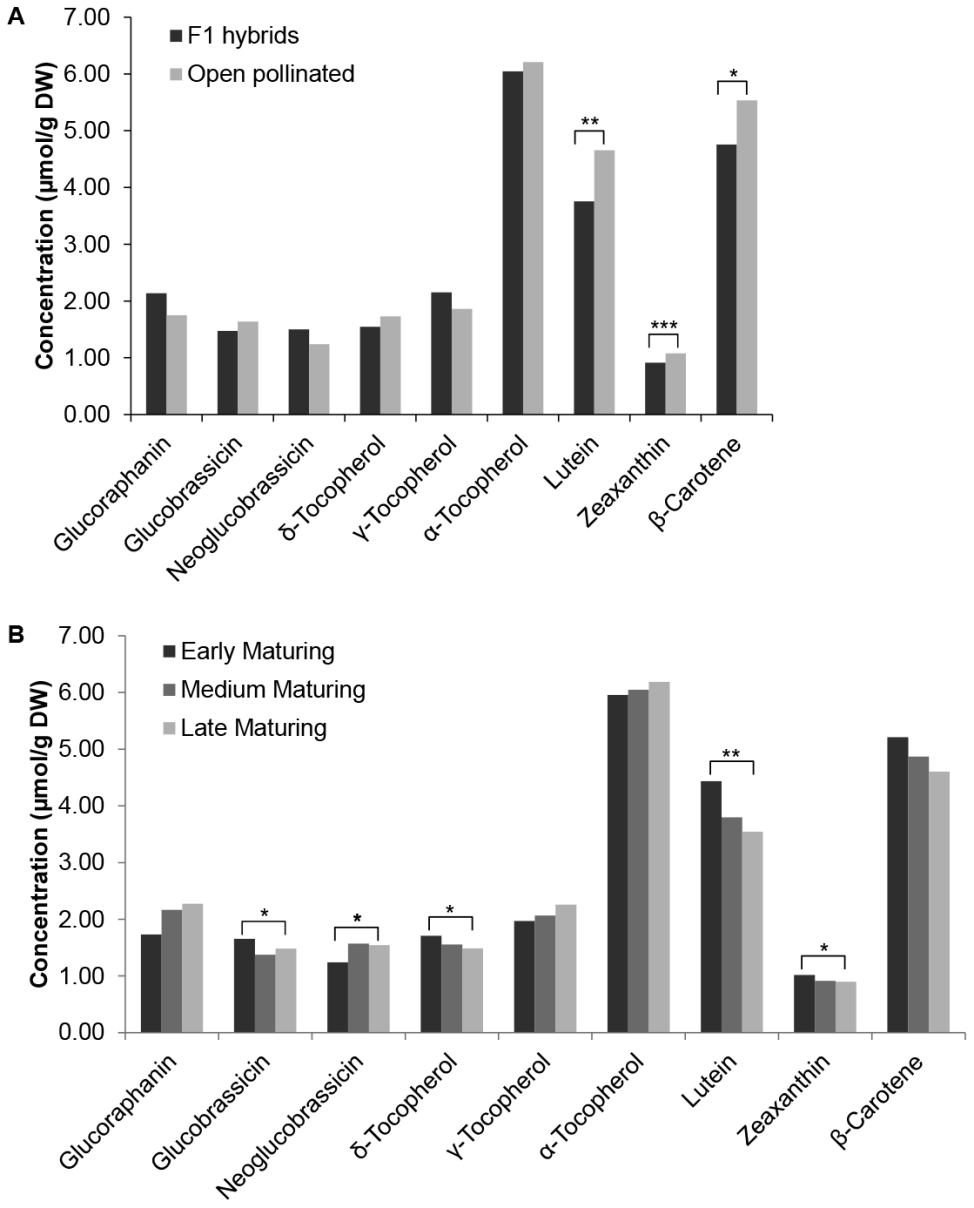
Mean phytochemical content of broccoli genotypic classes. A. Mean phytochemical content of broccoli F_1_ hybrids versus open pollinated cultivars, and B. Mean phytochemical content of early, mid- and late-maturing cultivars grown across all trials at two locations (Maine and Oregon), in two seasons (Fall and Spring) and in two management systems (Conventional and Organic) and conventional management systems. See Table 1 for key to cultivar F1 hybrid versus open pollinated classification and maturity classification. Significance (*  = P<0.05, **  = P<0.01, ***  = P<0.001).

The open pollinated and F_1_ hybrid cultivars were compared across trials for each phytochemical analysed (**Figure 2A**). The levels of glucoraphanin in F_1_ hybrids tended to be higher than the open pollinated cultivars. But the inverse trend was observed for glucobrassicin, which was supported by the ranking and stability analysis where the F_1_ hybrids showed higher levels and more stability across trials than the open pollinated cultivars for glucoraphanin. The reverse was observed for glucobrassicin. For the carotenoids, the open pollinated cultivars had a significantly higher mean value of lutein and zeaxanthin and tended to be higher for β-carotene compared to the F_1_ hybrids.

Based on the results of our field trials, the 23 cultivars of broccoli were grouped into three distinct maturity classes: Early (55–63 days); Mid (64–71 days); and Late (72–80 days) and analysed for the effect of the maturity class on phytochemical content (**Figure 2B**). For glucoraphanin, late maturing cultivars had significantly higher content levels, while for the carotenoids, early maturing cultivars tended to have higher concentrations and were significantly higher for lutein.

When cultivar performance between genetic material originating from two primary broccoli breeding companies was compared for phytochemical content there were no significant differences with the exception of lutein, where company 1's cultivars had significantly higher concentrations than those of company 2 (**data not shown**).

**Figure 3:**
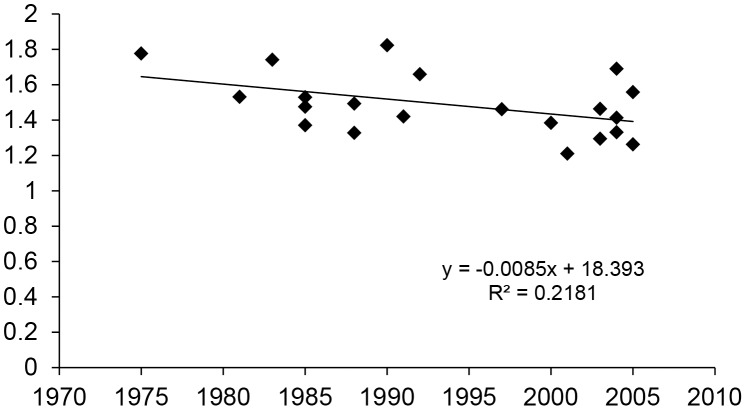
Regression of broccoli floret glucobrassicin concentrations on date of cultivar release for 23 cultivars grown across all trials in two locations (Maine and Oregon), in two seasons (Fall and Spring), in two management systems (Conventional and Organic), 2006–2008.

A negative correlation between the date of release and levels of glucobrassicin (R^2^ = 0.21; p = 0.03) (**Figure 3**) was observed, but no significant correlations for any other phytochemical were seen when 21 cultivars (the total set minus the two inbred lines) were analysed by their date of commercial release (1975–2005).

### Correlation analysis among phytochemicals and horticulture traits

#### Phytochemical correlation across trials

Correlation among phytochemicals indicated that glucoraphanin was significantly negatively correlated to glucobrassicin (**Table 5**). Correlations between the glucosinolates and the tocopherols were not significant. Glucoraphanin and neoglucobrassicin were negatively correlated to all carotenoids but only lutein and glucoraphanin were statistically significant. Glucobrassicin demonstrated a positive trend with all carotenoids. No statistically significant correlations were observed within tocopherols. Δ-tocopherol was positively correlated, while γ-tocopherol was negatively correlated to all carotenoids. There were no significant correlations for α-tocopherol with carotenoids. All carotenoids were highly positively correlated with one another.

**Table 5:** Correlations coefficients (r) for six horticultural traits and nine phytochemicals, calculated using data standardized across trials.

	Head Weight	Head Diameter	Maturity	Head Color	Bead Size	Bead Uniformity	Glucoraphanin	Glucobrassicin	Neoglucobrassicin	δ-tocopherol	γ-tocopherol	α-tocopherol	Lutein	Zeaxanthin	β-Carotene
Head Weight															
Head Diameter	0.81														
Maturity															
Head Color															
Bead Size	0.63		0.69												
Bead Uniformity	0.49	0.48													
Glucoraphanin	0.47	0.44	0.43		0.63	0.51									
Glucobrassicin	−0.54	−0.50			−0.56	−0.64	−0.51								
Neoglucobrassicin			0.58		0.48										
δ-Tocopherol	−0.55			0.49											
γ-Tocopherol					0.43										
α-Tocopherol															
Lutein	−0.65		−0.70	0.56	−0.69		−0.41			0.55	−0.54				
Zeaxanthin	−0.68	−0.43	−0.62	0.49	−0.64					0.60	−0.42		0.95		
β-Carotene	−0.53		−0.54	0.59	−0.48					0.50	−0.43		0.90	0.90	

Correlation results include means from 23 cultivars, across eight pair combinations of location (Maine/Oregon), season (Fall/Spring) and management system (Conventional/Organic), 2006–2008^a^.

a For empty cells, r is not significantly different from zero (P<0.05).

#### Phytochemical correlation to horticulture traits across trials

A correlation analysis was conducted for six horticulture traits, derived from the field study component of this research, Renaud et al. [78], and the nine phytochemicals across trials. The results indicated that greater head weight and head diameter were significantly positively correlated with glucoraphanin and negatively correlated with glucobrassicin, δ-tocopherol and the carotenoids. Increasing days to maturity was positively correlated with glucoraphanin, and negatively correlated to carotenoids. Head color was significantly correlated with δ-tocopherol and the carotenoids, but not with glucosinolates or γ- and α-tocopherol. Bead size and bead uniformity were positively correlated with glucoraphanin, neoglucobrassicin and γ-tocopherol and negatively correlated with glucobrassicin and the carotenoids.

### Principal component biplot analysis: correlation between phytochemicals and cultivars by production system

**Figure 4:**
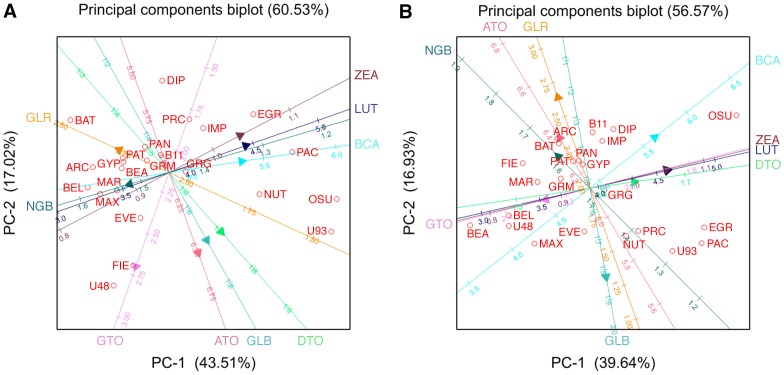
Principal components biplot of phytochemicals (vectors) and 23 cultivars (circles) grown in four seasons in Oregon and Maine. A. Biplot for conventional production, B. Biplot for organic production. See Table 1 for cultivar name abbreviations. Trait abbreviations: GLR: Glucoraphanin; GLB: Glucobrassicin; NGB: Neoglucobrassicin; DTO: δ-tocopherol; GTO: γ-tocopherol; ATO: α-tocopherol; LUT: Lutein; ZEA: Zeaxanthin; BCA: β-Carotene.

In the principal component analysis the first PC axis accounted for similar amounts of the total variation in both conventional and organic production systems (43.5% vs. 39.6%). The second PC axis showed a similar trend with 17.02% for conventional and 16.93% for organic (**Figures 4A and 4B**). The first two PC axes together accounted for 60.53% and 56.57% of total variation for conventional and organic, respectively. The PCA biplot analysis supported our findings that carotenoids were highly associated across systems, while tocopherols were highly associated in conventional, but not in organic (tocopherols demonstrated the largest shift between production systems). Glucoraphanin and neoglucobrassicin were associated with one another, but not with glucobrassicin across production systems. Glucoraphanin was associated with α-tocopherol in organic, but not in conventional treatments. Glucobrassicin was associated with δ- and α-tocopherol in conventional, but not in organic treatments. δ-tocopherol had a higher association with the carotenoids in organic than conventional. The biplots show response of both cultivars and phytochemical traits to environment. Those cultivars close to the origin reveal little about the relationship of cultivars and trait vectors, whereas those located near the extremes of trait vectors are those with the highest (or lowest) values for those traits.

## Discussion

### Impact of organic management system compared to environmental factors on phytochemical content

Few studies have specifically compared the levels of health promoting compounds in *Brassica* vegetable species grown under organic and conventional production systems [13], [74]–[75]. To our knowledge, this investigation is the most comprehensive study with the broadest range of phytochemical compounds (9) and a diverse set of broccoli cultivars (23) over regions (2), and management systems (2), with Fall and Spring season trials (2 each). In this study organic versus conventional management systems contributed the smallest source of variation compared to genotype, region and season. Within the phytochemicals studied individual compound concentrations responded differently. All compounds showed genetic variation, but also a substantial proportion of variance components were accounted for by high level interactions (**Table 3** **; Table S2 in File S1**). While M main effect was generally small, it had a substantial contribution in three- and four-way interactions. In particular, many G×L×S×M interactions were large relative to other variance components. This indicates that for the phytochemicals, M did have an influence on G, but that there were no consistent patterns across locations and seasons that would have shown up as significant G×M. Rather in each season and location, the paired organic and conventional environments differed significantly from one another but each situation was unique. In contrast to many comparisons between organic and conventional production systems [79], it should be noted that in our trials, yields averaged over the years did not differ significantly between the organic and conventional management systems [78].

Among the nine compounds, glucoraphanin was the most strongly influenced by genotype followed by lutein: supporting the findings of several other broccoli studies where variation in concentrations for glucoraphanin [19], [70], [65]–[66] and lutein [21], [37] was primarily due to genotype. For γ-tocopherol, genotype was a large source of variation, but this compound was equally influenced by location and season (also found by Ibrahim and Juvik [37]). For glucobrassicin and neoglucobrassicin the location was the largest source of variation, but also L×S interaction was very influential, particularly for neoglucobrassicin, which is supported by Kushad et al. [20] and Schonhof et al. [26]. Jasmonic acid, a signal transduction compound in plants, is up-regulated under conditions of plant stress, wounding, and herbivory. Increased endogenous levels or exogenous application of this compound (or methyl jasmonate) increases biosynthesis and transport of neoglucobrassicin to broccoli florets. This up-regulation was not observed for glucobrassicin biosynthesis [17]. This could explain why neoglucobrassicin was primarily under the control of Location and L×S interaction in our study. Season was the largest variance component for δ-tocopherol and zeaxanthin, which contrasts with the work of Ibrahim and Juvik [37] who found genotype had the largest influence on these compounds, followed by genotype by environment interaction although this study was constrained by the fact that the experiment was conducted in only one location over two growing seasons. For the other compounds such as α-tocopherol and β-carotene, L×S and the G×L×S interactions were most important.

Overall we found high genetic correlations between glucosinolates in organic and conventional trials. When trial locations were analysed separately, M main effect was present for glucobrassicin and neoglucobrassicin. The mean concentrations of glucobrassicin and neoglucobrassicin in broccoli from Oregon organic trials had higher concentrations compared to Oregon conventional trials, while Maine trials were comparable between management systems (**Table 2** **, Figure S1**). These results can be explained by the larger environment effect on glucobrassicin and genotype by environment effect on neoglucobrassicin found in the variance component analysis indicating sensitivity of these compounds to abiotic and/or biotic stresses. Our location specific findings are supported by those of Meyer and Adam [13] who performed a comparative study of the glucosinolate content of store bought organic and conventional broccoli and determined that the indolyl glucosinolates, glucobrassicin and neoglucobrassicin were significantly higher in the organically grown versus the conventionally grown. Evaluation of 10 broccoli genotypes over two years by Brown et al. [19] further supports our findings and those of Rosa and Rodrigues [62], Vallejo et al. [64], and Farnham et al. [70], that variation in concentration for glucoraphanin was primarily due to genetic variation, while differences in glucobrassicin was due to environmental variation (e.g. season, temperature) and genotype by environment interaction. The significantly higher levels of glucobrassicin in Oregon in the Fall harvested trials compared to Maine could be attributed to the higher maximum temperatures and GDD in Oregon compared to Maine (**Table S1 in File S1**).

Compared to glucosinolates, there is substantially less research on the genotype by environment interaction of tocopherol and carotenoid phytochemical groups in broccoli, and no specific studies exploring the influence of organic production system. In our study, minor management system effect at the overall trial analysis level was observed for the tocopherols and for carotenoids, there was management system effect only for lutein in Oregon Spring trials. Picchi et al. [75] also did not find differences in levels of carotenoids in cauliflower in organic versus conventional systems. In the tocopherols, there were no significant differences in location, but for δ- and γ- tocopherol concentration levels were higher in the fall compared to the spring, while for α-tocopherol, concentration levels were higher in the spring compared to the fall. For the carotenoids, there were no significant location differences, however there was a seasonal trend that all carotenoids were higher in spring compared to fall. Ibrahim and Juvik [39] found significant environmental variation among 24 broccoli cultivars for carotenoids and tocopherols which they attributed to the stressful production environments. Factors explaining the genotype and genotype by environment interaction components of variation in the carotenoids and tocopherols could be clarified by the fact that environmental stimuli are both up- and down-regulating genes associated with carotenoid and tocopherol biosynthesis. There is evidence in the literature that there are coordinated responses of the carotenoid and tocopherol antioxidants *in vivo*. There was a reduction in rape seed (*Brassica napus*) tocopherol content in response to increased carotenoid levels due to over expression of the enzyme phytoene synthase [80]. This response could explain the negative correlation between γ-tocopherol concentration and the carotenoids observed in our trials.

### Differences in phytochemical content between different genotypes and genotypic classes

The partitioning of variance indicated that genotype was an important source of variation for all glucosinolates. The cultivar ranking and rank correlation analysis demonstrated that there was a pattern in genotype content of glucosinolates where cultivars with the highest concentrations of glucoraphanin had the lowest levels for glucobrassicin (**Figures S1**). In our trials, the range in glucoraphanin concentrations across cultivars was (1.15–7.02 µmol/g DW, **Table S4 in File S1**), while glucobrassicin was 1.46–3.89 µmol/g DW, **Table S5 in File S1**). Several of the cultivars with the highest concentrations of neoglucobrassicin were those that had the highest concentrations of glucobrassicin. Range in neoglucobrassicin concentrations across cultivars was 0.68–4.54 µmol/g DW, **Table S6 in File S1**). In earlier studies, glucosinolate concentrations in broccoli have shown dramatic variation among different genotypes. Rosa and Rodriguez [62] studied total glucosinolate levels in eleven cultivars of broccoli and found ranges from 15.2–59.3 µmol/g DW. Among 50 accessions of broccoli Kushad et al. [20] found glucoraphanin content ranges from 0.8–22 µmol/g DW with a mean concentration of 7.1 µmol/g DW, while Wang et al. [61] found glucoraphanin content of five commercial hybrids and 143 parent materials ranging from 1.57–5.95 µmol/g for the hybrids and 0.06–24.17 µmol/g in inbred lines and Charron et al. [65] found ranges from 6.4–14.9 µmol/g DW. While the means in our study are somewhat lower, they are within the range of other studies.

A genotype effect was observed for tocopherols, but predominantly for γ-tocopherol. The PCA biplots (**Figure 4AB**) and the correlation analysis (**Table 5**) demonstrated the high positive correlations between δ-tocopherol, α-tocopherol and the carotenoids (α-tocopherol and β-carotene were also highly correlated in the Kushad et al. [20] study). The cultivar relationship to different phytochemicals was represented in the biplots as well as in the cultivar content and stability analysis (**Figure 1**). Many cultivars with the highest concentrations in the tocopherols and carotenoids were open pollinated cultivars, inbreds and early maturing, older F_1_ hybrids. Many of this same group were also relatively high in glucobrassicin concentrations. Kurilich et al. [38] found that carotenoid and tocopherol concentrations among 50 broccoli lines were highly variable and primarily genotype dependent. Specifically, levels of β-carotene ranged from 0.4–2.4 mg/100 g FW. Ibrahim and Juvik [37] also found broad ranges for total carotenoid and tocopherol concentrations among 24 genotypes ranging from 55–154 µg/g DW and 35–99 µg/g DW, respectively. Farnham and Kopsell [21] studied the carotenoid levels of nine double haploid lines of broccoli. Similar to our findings, lutein was the most abundant carotenoid in broccoli ranging from 65.3–139.6 µg/g DM. The sources of variation for lutein were predominantly genotype, followed by environment and G×E interaction, which also supports our findings. No genotypic differences were found for β-carotene in Farnham and Kopsell [21], which is in contrast to our findings. Overall, they found that most of the carotenoids measured were positively and highly correlated to one another as was observed in our study (**Table 5**). Kopsell et al. [81] found lutein levels in kale of 4.8–13.4 mg/100 g FW where the primary variance components for both lutein and β-carotene were also genotype and season.

Our research aimed also to address the question whether the phytochemical content of broccoli cultivars is associated with certain genotypic classes, e.g. open pollinated vs. F_1_ hybrids; older vs. newer cultivar releases; and between commercial sources. Broccoli is typically a cross-pollinated, self-incompatible crop species and cultivars are either open pollinated and composed of heterogeneous genetically segregating individuals, or F_1_ hybrids produced by crossing of two homozygous inbred lines, resulting in homogeneous populations of heterozygous individuals. In the 1960's virtually all broccoli grown was derived from OPs. By the 1990's almost all commercial cultivars were hybrids [82].

In our trials with 18 F_1_ hybrids (released between 1975–2005) and 3 open pollinated cultivars (released from 1985–2005), we found several interesting trends related to genotype and genotypic class performance as it related to the three groups of phytochemicals. When analysing F_1_ hybrid and open pollinated cultivars, they also demonstrated different performance patterns depending upon the individual phytochemical or group of compounds analysed. When cultivars were ranked for content and stability per phytochemical, there were distinct trends for certain compounds such as late maturing, F_1_ hybrids outperforming early maturing F_1_ hybrids and open pollinated cultivars for glucoraphanin, while the inverse was found for glucobrassicin and all carotenoids studied. This analysis was further supported by the PCA biplots that showed a strong relationship for select cultivars to certain phytochemicals or groups of phytochemicals such as ‘OSU OP’ to the carotenoids. When the full set of cultivars was divided into F_1_ hybrid and open pollinated groups and the means compared by phytochemical, the results further supported the individual cultivar analysis where F_1_ hybrids had higher mean values for glucoraphanin than the open pollinated cultivars (**Figure 2A**). Clear cultivar performance differences were identified where early maturing versus late maturing cultivars performed differently depending upon the phytochemical (**Figure 2B**). We also found that late maturing cultivars had higher concentrations for glucoraphanin than early maturing lines (and the inverse for glucobrassicin and the carotenoids). Picchi et al. [75] studied the quantity of glucosinolates of an early and late maturing cultivar of cauliflower grown in one conventional and three organic production systems, and found a significantly higher level of glucoraphanin in the later maturing cultivar compared to the early maturing cultivar in the organic production system. Another interesting trend was that cultivars with higher concentration levels for those phytochemicals whose expression is heavily influenced by environmental factors were not necessarily the most stable across trial environments; as was the case with neoglucobrassicin, δ- and γ-tocopherol in our study. For traits where genotype played a more significant role in contributing to variation, cultivars with a higher concentration level tended to also be those that were most stable across environments as was seen for lutein and glucoraphanin concentrations.

No significant differences were found for cultivar performance in phytochemical concentrations between genetic materials originating from two distinct commercial sources, with the exception of lutein (data not shown). When the full set of broccoli cultivars were analyzed for a correlation between date of release and mean level of phytochemical content across trials, no significant correlation was found with the exception of a negative trend for glucobrassicin (**Figure 3**). Our data does not support the idea that modern breeding for high yield performance and disease resistance necessarily leads to a trade-off in level of phytochemicals. Previous reports examining the relationship between year of release and performance had focussed on wheat vitamin and mineral content [83]–[85], and mineral content in broccoli [86]–[87]. However these authors did not study phytochemical content and their results were equivocal on the question on an innate biological trade-off between increased yield and nutritional content.

Not many studies have included two or more groups of phytochemicals. In our study with three phytochemical groups we found that phytochemicals demonstrating a negative correlation with one another (e.g. glucoraphanin with the carotenoids), showed an inverse cultivar response: e.g. cultivars with highest concentrations of glucoraphanin were the lowest in the carotenoids and vice versa. When both horticultural traits and phytochemicals were analysed for their phenotypic correlation, head weight was significantly and positively correlated with glucoraphanin and negatively correlated with δ- and α-tocopherol and the carotenoids. Farnham and Kopsell [21] explained that negative correlations may occur as a result of increased biomass accumulation in a certain genotype that is not accompanied by increased carotenoid production, effectively lowering the carotenoid concentration in the immature broccoli florets when pigments are expressed. Comparatively, head color was highly correlated to the carotenoids and negatively correlated to the glucosinolates overall. The cultivar ‘OSU OP’ was explicitly bred for a dark green stem and head color, not only for a darker green dome surface but also for a dark green interior color between the florets of the dome and in the stem (personal communication, Jim Myers 2013). ‘OSU OP’ was the highest in overall carotenoid concentrations across trials as it is known that carotenoids are correlated with chlorophyll concentrations and the intensity of green pigmentation [88].

### Perspectives on breeding broccoli for enhanced phytochemical content specifically for organic agriculture

Our study included predominantly broccoli cultivars selected for broad adaptability in conventional production systems and not purposely bred for high phytochemical content nor for adaptation to organic agriculture. What we can conclude from our data is that there has been little change in levels of several phytochemicals over three decades of breeding. This may indicate genetic variation for phytochemicals is limited in elite germplasm, or it may be the result of the lack of selection tools for these traits. This may be changing with recent efforts to introgress high glucoraphanin from *B. villosa* to produce the high-glucoraphanin F_1_ cultivar ‘Beneforté’ [89]–[90], [44]. The seed industry needs to exploit known sources of variation in the genus *Brassica* to enhance levels of other health-promoting phytochemicals and to broaden the genetic diversity of commercial broccoli germplasm. Our finding of a strong correlation between dark green color and high carotenoid levels provides breeders with a simple and efficient means of increasing carotenoids. The three groups of phytochemicals studied contribute to health promotion in different ways. As these groups are related to different metabolic pathways selecting for one compound does not necessarily inadvertently improve the other compounds, and may even result in negative correlation as we have seen in our data between glucoraphanin and the carotenoids. Although these compounds belong to different metabolic pathways, their production may be coordinated through regulatory feedback loops, or the structural and/or regulatory genes controlling these pathways may be genetically linked.

Designing a breeding program for broccoli high in glucosinolates would require the following considerations generated from our research: (1) Glucoraphanin is a highly genetically determined compound with minor location and season main effects but with substantial G×L×S interaction. (2) Comparatively, glucobrassicin and neoglucobrassicin are more impacted by location and season and L×S interaction with highest glucobrassicin concentrations and largest range in our Oregon Fall trials and neoglucobrassicin highest in Oregon Spring trials. (3) Cultivar performance for glucoraphanin and glucobrassicin and neoglucobrassicin was negatively correlated indicating that there may be a trade-off between glucoraphanin on the one hand, and glucobrassicin and neoglucobrassicin on the other hand. (4) Selection for glucoraphanin without consideration of horticultural traits would probably result in larger headed and later maturing cultivars. Conversely, selection for smaller headed, early maturing cultivars would favor glucobrassicin and neoglucobrassicin at the expense of glucoraphanin.

A breeding program for broccoli for high tocopherol content would require: (1) Overall the tocopherols were more season, location and L×S dependent and had lower overall repeatabilities compared to the glucosinolates. In a structured genetic population where additive genetic variance could be partitioned, narrow sense heritability would likely be low, and increasing tocopherol content would best be conducted with breeding methods suited to low heritability traits; (2) δ- and γ-tocopherols were both season dependent and fall grown broccoli had higher concentrations of these compounds across trials and a wider range of content levels, whereas levels of α-tocopherol were higher in spring but the range was comparable under both seasons. Thus, fall would be the preferred environment for breeding for these compounds; (3) there were no significant differences for location for δ- or γ-tocopherol, but the average levels of α-tocopherol levels were significantly higher in Oregon than Maine, suggesting greater potential for genetic gain in the Oregon environment.

If the goal is to design a breeding program for broccoli enhancing the levels of carotenoids it would require the following considerations: (1) For all three carotenoids studied, genotypic variation, particularly for lutein, was relatively more important than location and season; (2) however, zeaxanthin exhibited a large S (spring) and L×S interaction. For both β-carotene and lutein, spring grown broccoli had significantly higher levels than fall produced. Thus, selection for carotenoids would probably be more effective in spring than in fall; (3) early maturing and small headed cultivars had higher levels of carotenoids. Since most of the carotenoids are associated with the outer surfaces of the inflorescence, smaller broccoli heads with a greater surface area to volume ratio should show higher concentrations of these compounds; (4) because carotenoids have high G main effect good germplasm sources as indicated in **Figure 1** have high concentrations of carotenoids and demonstrated stability across environments. As all three carotenoids are highly correlated with one another, selecting for one should effectively select for all; (5) selection for darker green colour more widely distributed throughout the tissues of the head should allow the breeder to relatively efficiently increase carotenoid content in broccoli.

In closing, we want to address the question of selecting in an organic or a conventional environment. The argument commonly used to support selecting in productive environments is that heritabilities are higher compared to resource poor environments [91]–[92]. Organic is often considered a low-external input environment, resulting on average in 20% less yield compared to conventional production [79]. Nevertheless, in our trials repeatabilities for some phytochemicals were higher or comparable to conventional (**Table 3**). Narrow sense heritabilities would be expected to be significantly lower. For those traits where repeatabilities were higher or comparable, direct selection under organic systems could enhance selection gain. In all cases, the ratio of correlated response to direct response was less than one suggesting that direct selection would allow more rapid progress than correlated selection. Our data on phytochemicals did not show a wider range of levels under organic conditions as we found for horticultural traits in the same trials [78], however, in several cases, repeatabilities in organic production were higher than in conventional.

To maximize efficiency in a breeding program, commercial breeders may seek to combine breeding for both conventional and organic markets, and a combination of strategies can be proposed. Some studies that utilized highly heritable (agronomic) traits, where cultivar yield performance ranked similarly between organic and conventional management systems and which had high genetic correlations, suggested that early breeding be conducted under conventional conditions, with the caveat that advanced breeding lines be tested under organic conditions for less heritable traits (e.g. Löschenberger et al. [93]; Lorenzano and Bernardo, 2008) [52]. In studies where cultivar yield performance differed between management systems and there were significant differences in cultivar ranking, and in some cases low genetic correlations for lower heritability traits (e.g. Kirk et al. [94]; Murphy et al. [48]), these studies recommended that cultivars intended for organic agriculture be selected only under organic conditions. In our study of phytochemicals, we would recommend for organic purposes selection under organic conditions for the compounds where genetic correlations between organic and conventional were moderate.

## Supporting Information

Figure S1 **Comparison of broccoli cultivars for glucosinolates (µmol/g DW) grown across all trials in two locations (Maine and Oregon), in two seasons (Fall and Spring), in two management systems (Conventional and Organic), and at the individual trial level, 2006–2008.** A. Glucoraphanin, B. Glucobrassicin, C. Neoglucobrassicin.
(TIF)

Figure S2 **Comparison of broccoli cultivars for tocopherols (µmol/g DW) grown across all trials in two locations (Maine and Oregon), in two seasons (Fall and Spring), in two management systems (Conventional and Organic), and at the individual trial level, 2006–2008.** A. δ-tocopherol, B. γ-tocopherol, C. α-tocopherol.
(TIF)

Figure S3 **Comparison of broccoli cultivars for carotenoids (µmol/g DW) grown across all trials in two locations (Maine and Oregon), in two seasons (Fall and Spring), in two management systems (Conventional and Organic), and at the individual trial level, 2006–2008.** A. Lutein, B. Zeaxanthin, and C. β-carotene.
(TIF)

File S1 **Supporting tables. Table S1.** Description of agronomic and environmental factors of the trial locations with paired organically and conventionally managed fields, 2006–2008. **Table S2.** Partitioning (%) of variance components for various traits of 23 broccoli cultivars grown across four pair combinations in Maine, season (Fall/Spring) and management system (Conventional/Organic), 2006–2008. Variance components reported as coefficients of variation. **Table S3.** Repeatability for broccoli for phytochemicals and per trial of 23 broccoli cultivars grown across eight pair combinations of location (Maine/Oregon), season (Fall/Spring) and management system (Conventional/Organic), 2006–2008. **Table S4.** Glucoraphanin level (µmol/g DW) of 23 cultivars grown under conventional (C) and organic (O) conditions in two locations (Maine and Oregon) in two seasons (Fall and Spring) from 2006–2008. **Table S5.** Glucobrassicin level (µmol/g DW) of 23 cultivars grown under conventional (C) and organic (O) conditions in two locations (Maine and Oregon) in two seasons (Fall and Spring) from 2006–2008. **Table S6.** Neoglucobrassicin level (µmol/g DW) of 23 cultivars grown under conventional (C) and organic (O) conditions in two locations (Maine and Oregon) in two seasons (Fall and Spring) from 2006–2008. **Table S7.** δ-tocopherol level (µmol/g DW) of 23 cultivars grown under organic (O) and conventional (C) conditions in two locations (Maine and Oregon) in two seasons (Fall and Spring) from 2006–2008. **Table S8.** γ-tocopherol level (µmol/g DW) of 23 cultivars grown under conventional (C) and organic (O) conditions in two locations (Maine and Oregon) in two seasons (Fall and Spring) from 2006–2008. **Table S9.** α-tocopherol level (µmol/g DW) of 23 cultivars grown under conventional (C) and organic (O) conditions in two locations (Maine and Oregon) in two seasons (Fall and Spring) from 2006–2008. **Table S10.** Lutein level (µmol/g DW) of 23 cultivars grown under conventional (C) and organic (O) conditions in two locations (Maine and Oregon) in two seasons (Fall and Spring) from 2006–2008. **Table S11.** Zeaxanthin level (µmol/g DW) of 23 cultivars grown under conventional (C) and organic (O) and conditions in two locations (Maine and Oregon) in two seasons (Fall and Spring) from 2006–2008. **Table S12.** β-carotene level (µmol/g DW) of 23 cultivars grown under conventional (C) and organic (O) conditions in two locations (Maine and Oregon) in two seasons (Fall and Spring) from 2006–2008.
(DOCX)
