# Supplementary figures and images for: Variation in Broccoli Cultivar Phytochemical Content under Organic and Conventional Management Systems: Implications in Breeding for Nutrition
Source: PLoS One. 2014 Jul 16;9(7):e95683. doi: 10.1371/journal.pone.0095683 (PMC4100739; doi:10.1371/journal.pone.0095683)

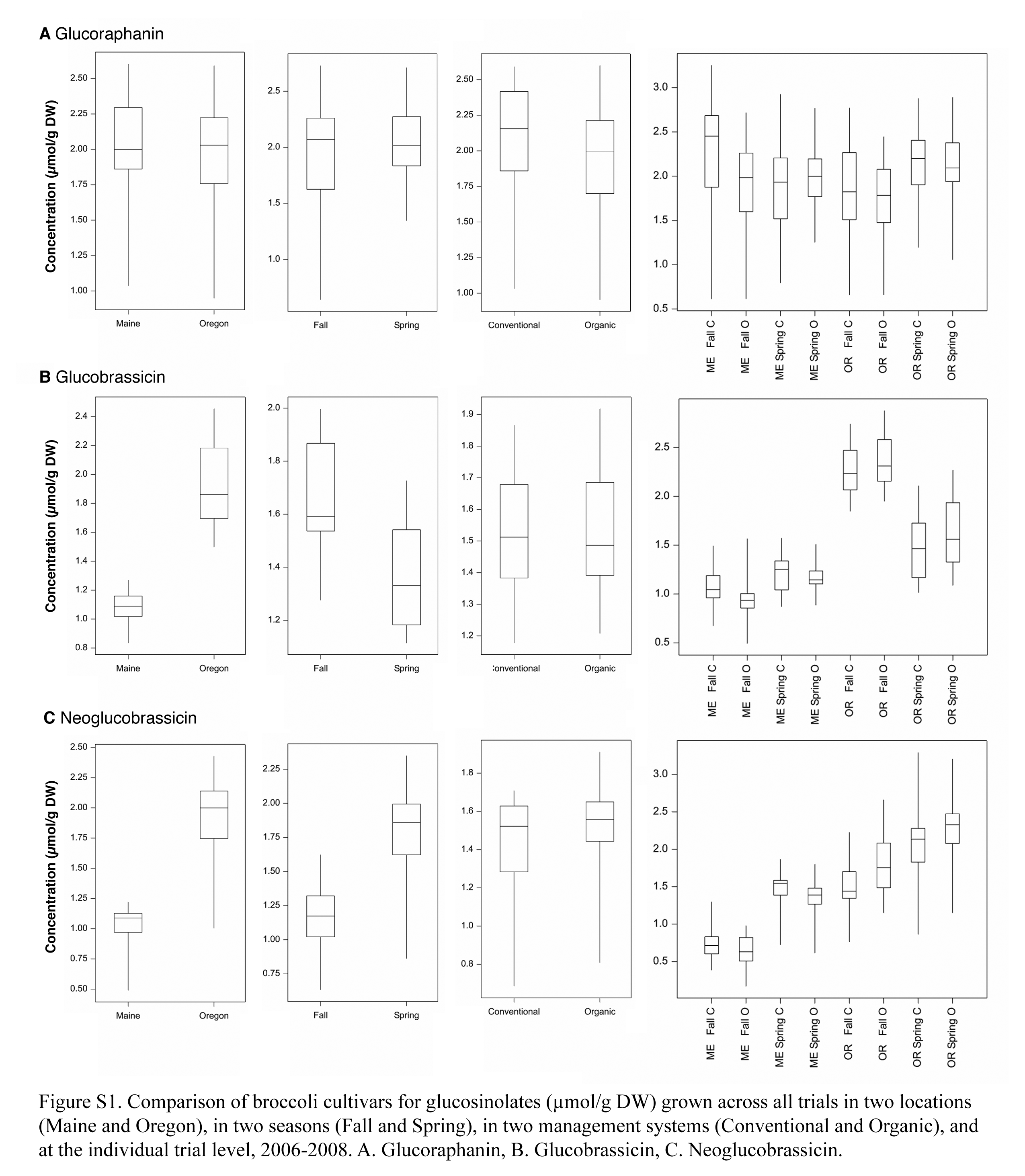

Supplement: Figure S1 — Comparison of broccoli cultivars for glucosinolates (µmol/g DW) grown across all trials in two locations (Maine and Oregon), in two seasons (Fall and Spring), in two management systems (Conventional and Organic), and at the individual trial level, 2006–2008. A. Glucoraphanin, B. Glucobrassicin, C. Neoglucobrassicin. (TIF) [file pone.0095683.s001.tif]

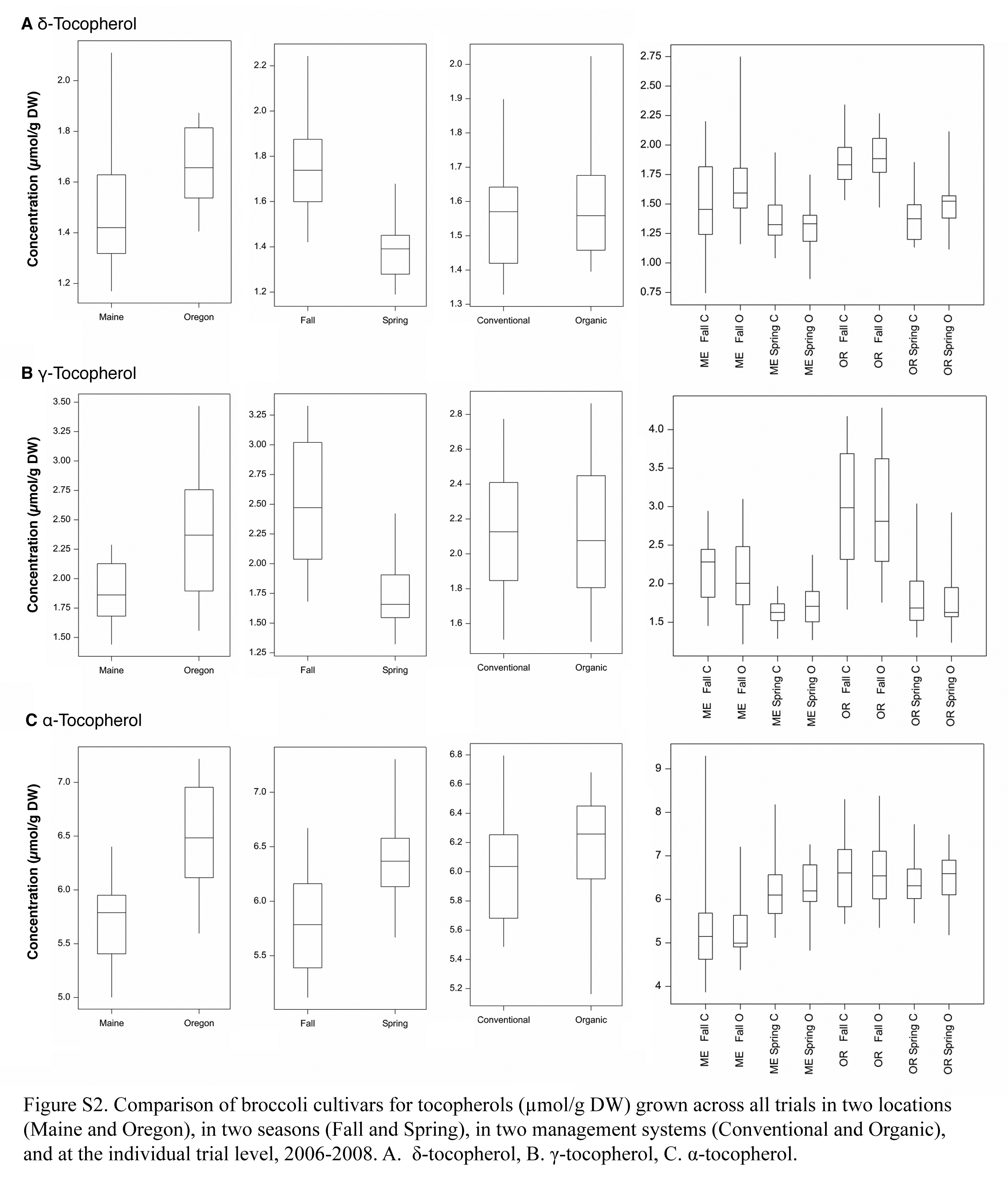

Supplement: Figure S2 — Comparison of broccoli cultivars for tocopherols (µmol/g DW) grown across all trials in two locations (Maine and Oregon), in two seasons (Fall and Spring), in two management systems (Conventional and Organic), and at the individual trial level, 2006–2008. A. δ-tocopherol, B. γ-tocopherol, C. α-tocopherol. (TIF) [file pone.0095683.s002.tif]

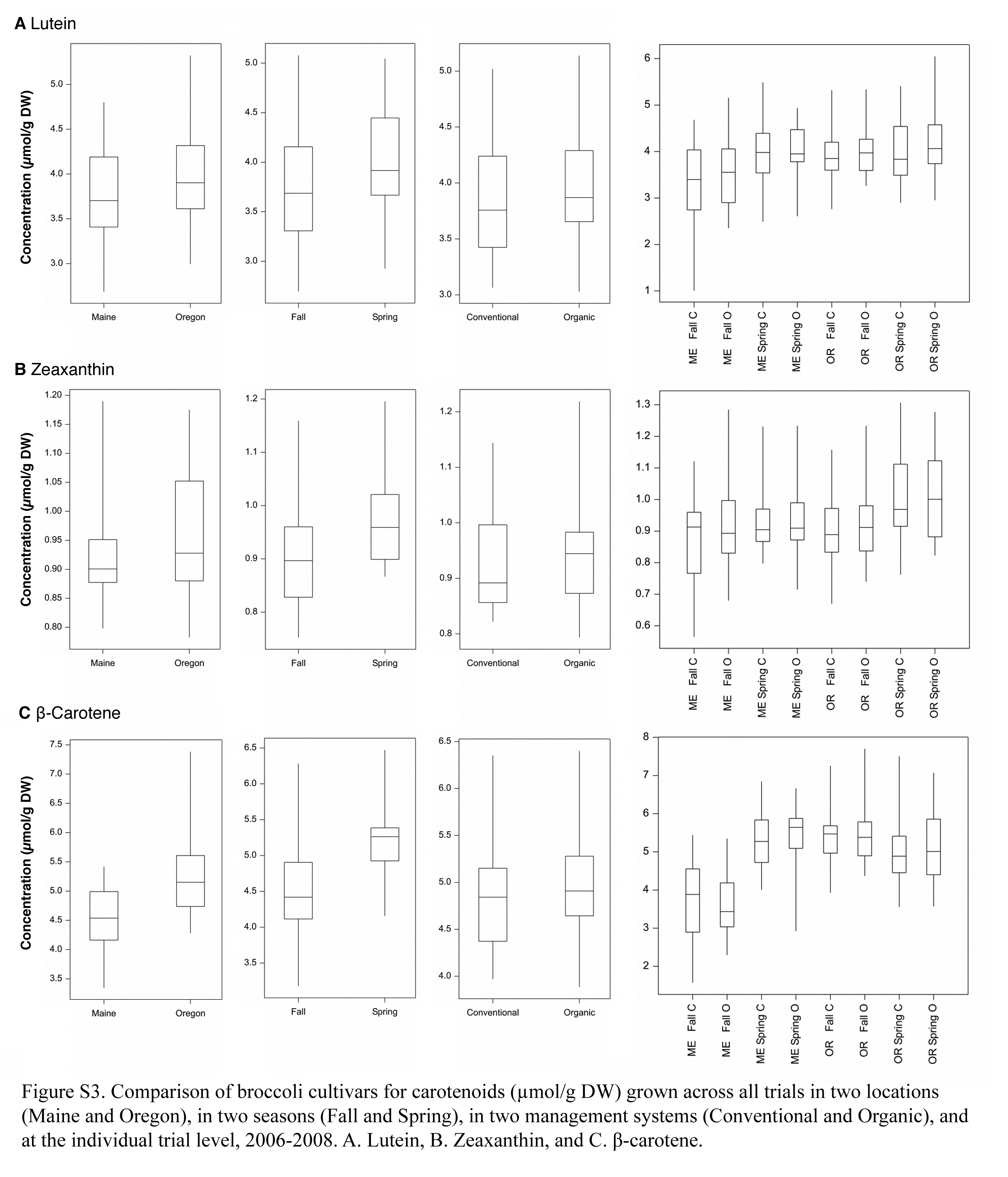

Supplement: Figure S3 — Comparison of broccoli cultivars for carotenoids (µmol/g DW) grown across all trials in two locations (Maine and Oregon), in two seasons (Fall and Spring), in two management systems (Conventional and Organic), and at the individual trial level, 2006–2008. A. Lutein, B. Zeaxanthin, and C. β-carotene. (TIF) [file pone.0095683.s003.tif]
