# Supplementary material for: Variation in Broccoli Cultivar Phytochemical Content under Organic and Conventional Management Systems: Implications in Breeding for Nutrition
Source: PLoS One. 2014 Jul 16;9(7):e95683. doi: 10.1371/journal.pone.0095683 (PMC4100739; doi:10.1371/journal.pone.0095683)
Supplement: File S1 — Supporting tables. Table S1. Description of agronomic and environmental factors of the trial locations with paired organically and conventionally managed fields, 2006–2008. Table S2. Partitioning (%) of variance components for various traits of 23 broccoli cultivars grown across four pair combinations in Maine, season (Fall/Spring) and management system (Conventional/Organic), 2006–2008. Variance components reported as coefficients of variation. Table S3. Repeatability for broccoli for phytochemicals and per trial of 23 broccoli cultivars grown across eight pair combinations of location (Maine/Oregon), season (Fall/Spring) and management system (Conventional/Organic), 2006–2008. Table S4. Glucoraphanin level (µmol/g DW) of 23 cultivars grown under conventional (C) and organic (O) conditions in two locations (Maine and Oregon) in two seasons (Fall and Spring) from 2006–2008. Table S5. Glucobrassicin level (µmol/g DW) of 23 cultivars grown under conventional (C) and organic (O) conditions in two locations (Maine and Oregon) in two seasons (Fall and Spring) from 2006–2008. Table S6. Neoglucobrassicin level (µmol/g DW) of 23 cultivars grown under conventional (C) and organic (O) conditions in two locations (Maine and Oregon) in two seasons (Fall and Spring) from 2006–2008. Table S7. δ-tocopherol level (µmol/g DW) of 23 cultivars grown under organic (O) and conventional (C) conditions in two locations (Maine and Oregon) in two seasons (Fall and Spring) from 2006–2008. Table S8. γ-tocopherol level (µmol/g DW) of 23 cultivars grown under conventional (C) and organic (O) conditions in two locations (Maine and Oregon) in two seasons (Fall and Spring) from 2006–2008. Table S9. α-tocopherol level (µmol/g DW) of 23 cultivars grown under conventional (C) and organic (O) conditions in two locations (Maine and Oregon) in two seasons (Fall and Spring) from 2006–2008. Table S10. Lutein level (µmol/g DW) of 23 cultivars grown under conventional (C) and organic (O) conditions in two l [file pone.0095683.s004.docx]

**Table S1.** Description of agronomic and environmental factors of the trial locations with paired organically and conventionally managed fields, 2006-2008.

|  |  |  |  | **Field Management** | | | | |
| --- | --- | --- | --- | --- | --- | --- | --- | --- |
| **Location** | **Season / Year** | **Planting Date** | **Harvest End Date** | **Preceeding Crop** | **Organic Fertilizer** |  | **Conventional Fertilizer** | |
|  |  |  |  |  | **Type** | **Application** | **Type**  (N-P_2_O_5_-K_2_O) | **Application** |
| Oregon | Fall 2006 | 28/Jul | 13/Oct | Lettuce | Chicken Manure; Oakleaf Compost; Gypsum | 2,500 kg/ha;22.200 kg/ha;5,000kg/ha | 12-29-10 | 504 kg/ha^-1^ |
| Maine | Fall 2006 | 1/Aug | 19/Oct | Cucurbits | Manure Compost | 26,900 kg ha^-1^ | 10-10-10 | 560 kg/ha^-1^ |
| Oregon | Spring 2007 | 10/May | 23/Jul | Lettuce | Chicken Manure; Oakleaf Compost; Gypsum | 2,500 kg/ha;22.200 kg/ha;5,000kg/ha | 12-29-10 | 504 kg/ha^-1^ |
| Maine | Spring 2007 | 31/May | 22/Aug | Cucurbits | Manure Compost | 26,900 kg ha^-1^ | 10-10-10 | 560 kg/ha^-1^ |
| Oregon | Fall 2007 | 7/Aug | 20/Nov | Lettuce | Chicken Manure; Oakleaf Compost; Gypsum | 2,500 kg/ha;22.200 kg/ha;5,000kg/ha | 12-29-10 | 504 kg/ha^-1^ |
| Maine | Fall 2007 | 14/Aug | 20/Nov | Cucurbits | Manure Compost | 26,900 kg ha^-1^ | 10-10-10 | 560 kg/ha^-1^ |
| Oregon | Spring 2008 | 14/May | 21/Jul | Lettuce | Chicken Manure; Oakleaf Compost; Gypsum | 2,500 kg/ha;22.200 kg/ha;5,000kg/ha | 12-29-10 | 504 kg/ha^-1^ |
| Maine | Spring 2008 | 18/Jun | 10/Nov | Cucurbits | Manure Compost | 26,900 kg ha^-1^ | 10-10-10 | 560 kg/ha^-1^ |

**Table S1.** Description of agronomic and environmental factors of the trial locations with paired organically and conventionally managed fields, 2006-2008 (continued).

|  |  | **Field Management** | | **Soils** | | | | | | **Climate** | | | |
| --- | --- | --- | --- | --- | --- | --- | --- | --- | --- | --- | --- | --- | --- |
| **Location** | **Season / Year** | **Supplemental Irrigation (mm)** | | **pH** |  | **g POM-N/kg soil^a^** | | **Available N (ppm)^b^** | | **Average Temp** | | **Growing Degree Days^c^** | **Precipitation^d^** |
|  |  | **Org** | **Conv** | **Org** | **Conv** | **Org** | **Conv** | **Org** | **Conv** | **Max (^o^C)** | **Min (^o^C)** | **(Base Temp 4.4^o^C)** | **(mm)** |
| Oregon | Fall 2006 | 170.1 | 171.5 | 6.7 | 6.1 | 0.53 | 0.07 | 1.17 | 1.22 | 25.5 | 8.2 | 1741 | 48.3 |
| Maine | Fall 2006 | None | None | 6.7 | 5.7 | 0.44 | 0.54 | 1.43 | 1.63 | 20.8 | 10.4 | 1611 | 279.4 |
| Oregon | Spring 2007 | 131.9 | 135.4 | 6.4 | 6.5 | 0.35 | 0.06 | 1.14 | 1.48 | 24.2 | 9.0 | 1639 | 35.6 |
| Maine | Spring 2007 | None | None | 6.2 | 5.4 | 0.48 | 0.50 | 1.83 | 1.99 | 24.8 | 13.6 | 2212 | 167.6 |
| Oregon | Fall 2007 | 96.6 | 101.6 | 6.7 | 6.3 | 0.51 | 0.16 | 0.92 | 1.37 | 20.8 | 7.9 | 1823 | 274.3 |
| Maine | Fall 2007 | None | None | 6.2 | 5.4 | 0.44 | 0.44 | 1.16 | 1.24 | 18.0 | 7.6 | 1573 | 370.8 |
| Oregon | Spring 2008 | 157.2 | 156.5 | 6.9 | 6.4 | 0.64 | 0.11 | 1.55 | 1.75 | 24.1 | 9.2 | 1521 | 35.6 |
| Maine | Spring 2008 | None | None | 6.4 | 5.5 | 0.47 | 0.43 | 1.53 | 1.53 | 21.0 | 11.1 | 3059 | 581.7 |

^a^ g POM-N/kg soil: Particulate Organic Matter, a measure of longer term available nitrogen taken pre-fertilization.

^b^ Available N (ppm): immediately available nitrogen at time of trial planting taken pre-fertilization.

^c^ Growing Degree Days (GDD): average daily maximum and minimum temperatures across each trial period divided by base temperature for broccoli growth (4.4^o^C).

^d^ Total Precipitation during trial period per location, season not including irrigation.

**Table S2a.** Partitioning (%) of variance components for various traits of 23 broccoli cultivars grown across four pair combinations in Maine, season (Fall/Spring) and management system (Conventional/Organic), 2006-2008. Variance components reported as coefficients of variation.

|  | **Season (S)** | **Genotype (G)** | **Management (M)** | **S x M** | **S x M x Rep (R)** | **G x S** | **G x M** | **G x S x M** | **Residual** |
| --- | --- | --- | --- | --- | --- | --- | --- | --- | --- |
| Glucoraphanin | 10.89 | 14.15 | 0.00 | 15.35 | 0.73 | 19.82 | 0.00 | 17.44 | 12.35 |
| Glucobrassicin | 29.06 | 0.00 | 5.18 | 6.15 | 2.79 | 20.67 | 0.00 | 16.99 | 13.87 |
| Neoglucobrassicin | 66.18 | 10.67 | 6.10 | 7.01 | 6.32 | 21.51 | 0.01 | 14.19 | 17.74 |
| δ-tocopherol | 30.79 | 10.48 | 0.00 | 4.31 | 0.00 | 20.62 | 0.00 | 12.27 | 14.45 |
| γ-tocopherol | 13.49 | 5.74 | 0.00 | 9.18 | 2.40 | 20.60 | 0.00 | 12.49 | 13.01 |
| α-tocopherol | 15.58 | 3.60 | 0.00 | 0.01 | 1.54 | 8.74 | 0.00 | 9.99 | 9.38 |
| Lutein | 7.09 | 13.11 | 0.00 | 0.00 | 0.01 | 12.87 | 2.38 | 9.92 | 10.80 |
| Zeaxanthin | 13.05 | 7.77 | 0.01 | 0.00 | 0.00 | 7.97 | 0.00 | 8.59 | 11.06 |
| β-Carotene | 24.49 | 2.23 | 0.00 | 0.00 | 1.50 | 14.75 | 8.65 | 16.22 | 12.23 |

**Table S2b.** Partitioning (%) of variance components for various traits of 23 broccoli cultivars grown across four pair combinations in Oregon, season (Fall/Spring) and management system (Conventional/Organic), 2006-2008. Variance components reported as coefficients of variation.

|  | **Season (S)** | **Genotype (G)** | **Management (M)** | **S x M** | **S x M x Rep (R)** | **G x S** | **G x M** | **G x S x M** | **Residual** |
| --- | --- | --- | --- | --- | --- | --- | --- | --- | --- |
| Glucoraphanin | 8.71 | 17.80 | 0.00 | 5.52 | 2.16 | 13.82 | 0.00 | 11.39 | 11.49 |
| Glucobrassicin | 24.94 | 14.51 | 4.37 | 3.60 | 1.29 | 8.19 | 0.00 | 7.19 | 9.08 |
| Neoglucobrassicin | 18.66 | 17.25 | 7.14 | 8.42 | 3.92 | 13.31 | 0.00 | 12.33 | 14.14 |
| δ-tocopherol | 40.18 | 5.38 | 2.23 | 1.13 | 0.00 | 7.99 | 0.00 | 11.85 | 11.21 |
| γ-tocopherol | 27.58 | 18.18 | 0.00 | 0.01 | 1.77 | 19.52 | 0.01 | 9.85 | 11.21 |
| α-tocopherol | 0.01 | 5.13 | 0.00 | 0.01 | 1.00 | 10.67 | 0.00 | 6.72 | 8.51 |
| Lutein | 10.02 | 14.05 | 0.00 | 4.50 | 1.88 | 8.82 | 0.00 | 8.21 | 9.13 |
| Zeaxanthin | 11.28 | 10.08 | 0.01 | 0.00 | 0.68 | 5.84 | 2.41 | 8.29 | 11.58 |
| β-Carotene | 9.37 | 12.16 | 0.00 | 1.55 | 0.00 | 7.91 | 0.00 | 9.22 | 9.88 |

**Table S3.** Repeatability for broccoli for phytochemicals and per trial of 23 broccoli cultivars grown across eight pair combinations of location (Maine/Oregon), season (Fall/Spring) and management system (Conventional/Organic), 2006-2008.

|  | **Maine** | | | | | | | |
| --- | --- | --- | --- | --- | --- | --- | --- | --- |
|  | **Fall** | | | | **Spring** | | | |
|  | **2006** | | **2007** | | **2007** | | **2008** | |
|  | **C** | **O** | **C** | **O** | **C** | **O** | **C** | **O** |
| Glucoraphanin | 0.88 | 0.86 | 0.99 | 0.97 | 0.92 | 0.88 | 0.97 | 0.95 |
| Glucobrassicin | 0.93 | 0.96 | 0.95 | 0.95 | 0.93 | 0.89 | 0.89 | 0.83 |
| Neoglucobrassicin | 0.87 | 0.52 | 0.91 | 0.93 | 0.81 | 0.92 | 0.85 | 0.74 |
| δ-tocopherol | 0.96 | 0.96 | 0.87 | 0.76 | 0.85 | 0.72 | 0.59 | 0.85 |
| γ-tocopherol | 0.91 | 0.93 | 0.98 | 0.96 | 0.76 | 0.73 | 0.53 | 0.89 |
| α-tocopherol | 0.89 | 0.86 | 0.83 | 0.94 | 0.69 | 0.78 | 0.86 | 0.89 |
| Lutein | 0.97 | 0.98 | 0.94 | 0.92 | 0.85 | 0.82 | 0.95 | 0.79 |
| Zeaxanthin | 0.91 | 0.97 | 0.88 | 0.76 | 0.69 | 0.75 | 0.79 | 0.85 |
| β-Carotene | 0.98 | 0.99 | 0.88 | 0.93 | 0.73 | 0.84 | 0.81 | 0.96 |
|  |  |  |  |  |  |  |  |  |
|  | **Oregon** | | | | | | | |
|  | **Fall** | | | | **Spring** | | | |
|  | **2006** | | **2007** | | **2007** | | **2008** | |
|  | **C** | **O** | **C** | **O** | **C** | **O** | **C** | **O** |
| Glucoraphanin | 0.93 | 0.92 | 0.94 | 0.93 | 0.91 | 0.94 | 0.96 | 0.92 |
| Glucobrassicin | 0.89 | 0.89 | 0.90 | 0.90 | 0.94 | 0.92 | 0.93 | 0.92 |
| Neoglucobrassicin | 0.88 | 0.95 | 0.88 | 0.87 | 0.90 | 0.92 | 0.93 | 0.75 |
| δ-tocopherol | 0.62 | 0.21 | 0.90 | 0.91 | 0.81 | 0.89 | 0.91 | 0.74 |
| γ-tocopherol | 0.96 | 0.91 | 0.98 | 0.98 | 0.87 | 0.88 | 0.94 | 0.93 |
| α-tocopherol | 0.98 | 0.93 | 0.90 | 0.88 | 0.75 | 0.81 | 0.77 | 0.70 |
| Lutein | 0.91 | 0.96 | 0.92 | 0.89 | 0.91 | 0.91 | 0.93 | 0.95 |
| Zeaxanthin | 0.90 | 0.75 | 0.88 | 0.85 | 0.80 | 0.79 | 0.83 | 0.87 |
| β-Carotene | 0.90 | 0.96 | 0.89 | 0.90 | 0.89 | 0.90 | 0.92 | 0.88 |

**Table S4.** Glucoraphanin level (µ mol/g DW) of 23 cultivars grown under conventional (C) and organic (O) conditions in two locations (Maine and Oregon) in two seasons (Fall and Spring) from 2006-2008.

|  | **Maine** | | | | | | | | **Oregon** | | | | | | | | **Overall Cultivar Mean AHW** | **Overall Cultivar Mean C** | **Overall Cultivar Mean O** |
| --- | --- | --- | --- | --- | --- | --- | --- | --- | --- | --- | --- | --- | --- | --- | --- | --- | --- | --- | --- |
|  | **Fall** | | | | **Spring** | | | | **Fall** | | | | **Spring** | | | |  |  |  |
|  | **2006** | | **2007** | | **2007** | | **2008** | | **2006** | | **2007** | | **2007** | | **2008** | |  |  |  |
|  | **C** | **O** | **C** | **O** | **C** | **O** | **C** | **O** | **C** | **O** | **C** | **O** | **C** | **O** | **C** | **O** |  |  |  |
| Arcadia | 6.05 | 5.07 | 15.12 | 9.12 | 5.92 | 8.56 | 1.60 | 7.06 | 8.83 | 5.05 | 5.80 | 6.42 | 8.09 | 7.03 | 6.06 | 6.50 | 7.02 | 7.19 | 6.85 |
| B1 10 | 5.18 | 3.72 | 13.83 | 6.66 | 5.63 | 8.81 | 1.57 | 8.06 | 6.16 | 6.50 | 4.90 | 4.85 | 9.63 | 6.35 | 4.05 | 7.97 | 6.49 | 6.37 | 6.61 |
| Batavia |  |  | 4.92 | 2.13 | 2.49 | 4.69 | 8.95 | 4.14 |  |  | 2.31 | 2.58 | 5.84 | 6.19 | 1.70 | 2.38 | 4.03 | 4.37 | 3.69 |
| Beaumont |  |  |  | 5.93 | 4.88 | 3.28 | 2.42 | 2.89 |  |  | 3.17 | 2.40 | 6.97 | 7.33 | 3.09 | 1.55 | 3.99 | 4.11 | 3.90 |
| Belstar | 4.20 | 3.38 | 12.82 | 4.84 | 6.31 | 5.89 | 1.12 | 0.86 | 8.23 | 3.73 | 3.34 | 3.97 | 4.16 | 6.21 | 6.62 | 4.82 | 5.03 | 5.85 | 4.21 |
| Diplomat |  |  | 10.41 | 4.78 | 8.20 | 8.33 | 9.04 | 3.53 |  |  | 2.29 | 3.24 | 5.84 | 8.24 | 8.66 | 8.92 | 6.79 | 7.41 | 6.17 |
| Early Green | 2.88 | 3.80 | 2.99 | 0.82 | 1.94 | 2.54 | 1.56 | 1.67 | 2.02 | 1.18 | 1.29 | 1.69 | 3.98 | 2.43 | 2.20 | 2.43 | 2.21 | 2.36 | 2.07 |
| Everest | 2.41 | 5.03 | 4.58 | 0.40 | 3.26 | 5.50 |  |  | 2.09 | 1.64 | 1.43 | 2.12 | 4.79 | 5.26 | 2.84 | 2.57 | 3.14 | 3.06 | 3.22 |
| Fiesta | 4.60 | 4.43 |  | 5.81 | 5.80 | 5.61 | 3.75 | 9.22 | 5.69 |  | 4.61 | 5.04 | 12.22 | 8.54 | 5.38 | 6.28 | 6.21 | 6.01 | 6.42 |
| Green Goliath | 2.62 | 4.70 | 12.45 | 4.41 | 4.92 | 7.94 | 1.12 | 1.23 | 6.04 | 1.66 | 3.58 | 5.32 | 2.93 | 2.52 | 4.63 | 5.79 | 4.49 | 4.79 | 4.20 |
| Green Magic | 3.66 | 3.99 | 9.60 | 2.08 | 6.46 | 5.02 | 3.47 | 3.28 | 3.64 | 3.53 | 3.88 | 3.21 | 4.15 | 3.20 | 6.27 | 5.09 | 4.41 | 5.14 | 3.68 |
| Gypsy | 4.88 | 5.03 | 11.56 | 2.04 | 8.73 | 9.85 | 1.44 | 1.67 | 6.33 | 9.12 | 5.17 | 5.29 | 6.89 | 4.42 | 4.51 | 6.17 | 5.82 | 6.19 | 5.45 |
| Imperial |  |  |  | 5.33 |  | 10.13 | 0.31 | 2.40 |  |  | 2.81 | 3.42 | 3.99 | 5.39 | 4.76 | 7.68 | 4.62 | 2.97 | 5.73 |
| Marathon | 3.30 | 2.51 | 9.85 | 6.39 | 5.70 | 3.54 | 3.45 | 4.83 | 4.91 | 3.64 | 6.42 | 5.08 | 6.27 | 5.17 | 2.61 | 4.51 | 4.89 | 5.31 | 4.46 |
| Maximo | 2.35 | 2.36 | 9.42 | 4.64 | 7.79 | 6.82 | 1.89 | 1.97 | 3.10 | 2.47 | 4.39 | 4.19 | 7.31 | 6.13 | 2.91 | 3.14 | 4.43 | 4.90 | 3.97 |
| Nutribud | 3.40 | 4.01 | 3.28 | 1.10 | 2.37 | 5.73 | 2.62 | 1.97 | 2.15 | 2.25 | 1.71 | 1.97 | 2.17 | 2.33 | 0.81 | 3.33 | 2.58 | 2.31 | 2.84 |
| OSU OP | 10.25 | 10.64 | 3.55 | 1.86 | 5.92 | 7.05 | 6.59 | 2.43 | 2.64 | 2.35 | 4.65 | 5.16 | 6.72 | 7.23 | 2.70 | 4.59 | 5.27 | 5.38 | 5.16 |
| Packman | 3.40 | 4.08 | 2.25 | 1.36 | 3.61 | 4.48 | 2.88 | 1.51 | 1.01 | 1.36 | 2.27 | 4.62 | 4.89 | 2.73 | 1.53 | 1.74 | 2.73 | 2.73 | 2.73 |
| Patriot |  |  | 7.41 | 4.74 | 5.07 | 7.48 | 11.40 | 1.84 |  |  | 2.89 | 2.93 | 7.36 | 8.57 | 8.29 | 3.27 | 5.94 | 7.07 | 4.81 |
| Patron | 7.33 | 3.96 | 11.99 | 4.47 | 4.52 | 4.58 | 0.51 | 0.27 | 4.13 | 1.72 | 2.14 | 2.15 | 9.82 | 4.01 | 4.31 | 5.49 | 4.46 | 5.59 | 3.33 |
| Premium Crop | 6.52 | 6.15 | 5.51 | 1.39 | 7.39 | 7.01 | 1.84 | 1.03 | 3.84 | 1.99 | 1.46 | 1.92 | 5.50 | 2.96 | 4.43 | 4.14 | 3.94 | 4.56 | 3.32 |
| USVL 048 | 3.25 | 4.16 |  | 10.52 | 3.80 | 4.66 | 1.88 | 1.56 |  |  | 5.27 | 5.38 | 2.58 | 2.40 | 3.83 | 4.28 | 4.12 | 3.43 | 4.71 |
| USVL 093 | 0.10 | 0.30 | 0.67 | 0.55 | 1.12 | 2.91 | 1.96 | 1.27 |  |  | 0.47 | 0.43 | 1.44 | 0.64 | 2.30 | 1.99 | 1.15 | 1.15 | 1.16 |
|  |  |  |  |  |  |  |  |  |  |  |  |  |  |  |  |  |  |  |  |
|  |  |  |  |  |  |  |  |  |  |  |  |  |  |  |  | **Mean** | 4.51 | 4.71 | 4.29 |

**Table S5.** Glucobrassicin level (µ mol/g DW) of 23 cultivars grown under conventional (C) and organic (O) conditions in two locations (Maine and Oregon) in two seasons (Fall and Spring) from 2006-2008.

|  | **Maine** | | | | | | | | **Oregon** | | | | | | | | **Overall Cultivar Mean AHW** | **Overall Cultivar Mean C** | **Overall Cultivar Mean O** |
| --- | --- | --- | --- | --- | --- | --- | --- | --- | --- | --- | --- | --- | --- | --- | --- | --- | --- | --- | --- |
|  | **Fall** | | | | **Spring** | | | | **Fall** | | | | **Spring** | | | |  |  |  |
|  | **2006** | | **2007** | | **2007** | | **2008** | | **2006** | | **2007** | | **2007** | | **2008** | |  |  |  |
|  | **C** | **O** | **C** | **O** | **C** | **O** | **C** | **O** | **C** | **O** | **C** | **O** | **C** | **O** | **C** | **O** |  |  |  |
| Arcadia | 0.47 | 0.38 | 2.19 | 1.54 | 1.99 | 1.55 | 0.35 | 1.45 | 5.25 | 6.72 | 3.05 | 3.93 | 1.02 | 1.51 | 1.46 | 2.04 | 2.18 | 1.81 | 2.39 |
| B1 10 | 0.29 | 0.31 | 1.94 | 1.42 | 2.00 | 1.38 | 0.50 | 1.55 | 4.81 | 5.44 | 2.67 | 3.97 | 1.21 | 1.02 | 1.66 | 2.08 | 2.02 | 1.71 | 2.15 |
| Batavia |  |  | 0.87 | 0.62 | 1.13 | 1.15 | 1.16 | 1.24 |  |  | 2.98 | 2.72 | 1.17 | 1.54 | 0.80 | 2.07 | 1.46 | 1.35 | 1.56 |
| Beaumont |  |  |  | 2.32 | 1.68 | 1.85 | 0.59 | 0.74 |  |  | 3.35 | 2.73 | 2.04 | 2.00 | 2.68 | 3.51 | 2.13 | 2.07 | 2.19 |
| Belstar | 0.23 | 0.23 | 3.79 | 1.59 | 1.35 | 2.01 | 0.32 | 0.36 | 7.09 | 8.97 | 3.09 | 2.87 | 2.12 | 2.52 | 2.22 | 3.54 | 2.64 | 2.27 | 2.76 |
| Diplomat |  |  | 1.05 | 1.02 | 1.15 | 1.12 | 2.36 | 1.33 |  |  | 4.53 | 3.49 | 0.93 | 1.41 | 1.57 | 1.77 | 1.81 | 1.93 | 1.69 |
| Early Green | 1.28 | 1.14 | 1.55 | 0.74 | 1.74 | 2.42 | 1.38 | 0.57 | 4.99 | 4.14 | 3.70 | 5.51 | 2.18 | 2.58 | 2.44 | 4.49 | 2.55 | 2.28 | 2.70 |
| Everest | 0.44 | 0.34 | 1.07 | 0.63 | 2.21 | 2.15 |  |  | 5.48 | 5.94 | 3.69 | 5.07 | 2.51 | 2.83 | 3.75 | 3.86 | 2.86 | 2.45 | 2.97 |
| Fiesta | 0.40 | 0.42 |  | 2.36 | 2.29 | 1.97 | 1.57 | 1.80 | 8.44 |  | 4.60 | 4.43 | 3.56 | 1.32 | 3.05 | 3.97 | 2.87 | 3.04 | 2.32 |
| Green Goliath | 0.26 | 0.57 | 2.50 | 1.82 | 3.55 | 2.81 | 1.25 | 1.43 | 8.12 | 5.15 | 3.13 | 3.03 | 1.66 | 1.44 | 1.88 | 4.14 | 2.67 | 2.51 | 2.55 |
| Green Magic | 0.37 | 0.38 | 1.68 | 0.68 | 2.76 | 1.33 | 2.62 | 0.88 | 4.47 | 4.97 | 2.93 | 2.60 | 0.78 | 0.94 | 1.31 | 1.78 | 1.90 | 1.92 | 1.70 |
| Gypsy | 0.44 | 0.34 | 1.78 | 0.93 | 1.98 | 1.81 | 0.29 | 0.78 | 5.51 | 7.35 | 4.11 | 4.44 | 1.48 | 1.35 | 3.04 | 2.50 | 2.38 | 2.12 | 2.44 |
| Imperial |  |  |  | 1.48 |  | 1.30 | 0.45 | 0.52 |  |  | 2.65 | 2.79 | 1.28 | 1.87 | 1.40 | 2.21 | 1.60 | 1.44 | 1.70 |
| Marathon | 0.18 | 0.25 | 2.97 | 1.96 | 2.05 | 1.65 | 1.17 | 0.82 | 6.68 | 10.60 | 3.73 | 3.25 | 1.67 | 2.00 | 1.49 | 2.01 | 2.66 | 2.24 | 2.82 |
| Maximo | 0.24 | 0.43 | 4.02 | 1.74 | 2.44 | 2.71 | 0.42 | 0.45 | 8.32 | 10.21 | 6.46 | 6.31 | 2.37 | 4.05 | 2.13 | 3.79 | 3.51 | 2.96 | 3.71 |
| Nutribud | 0.91 | 0.88 | 3.23 | 0.76 | 3.25 | 3.62 | 0.59 | 0.46 | 8.84 | 8.02 | 5.79 | 6.69 | 4.28 | 3.14 | 4.68 | 7.09 | 3.89 | 3.61 | 3.83 |
| OSU OP | 1.36 | 0.90 | 0.90 | 0.49 | 2.17 | 2.71 | 1.47 | 0.43 | 4.93 | 6.67 | 3.87 | 4.63 | 3.67 | 3.93 | 2.43 | 3.33 | 2.74 | 2.46 | 2.89 |
| Packman | 2.07 | 1.20 | 1.40 | 1.05 | 2.91 | 1.95 | 0.48 | 0.46 | 5.55 | 7.71 | 4.56 | 7.71 | 6.56 | 4.15 | 3.31 | 4.72 | 3.49 | 3.21 | 3.62 |
| Patriot |  |  | 1.71 | 1.36 | 1.60 | 0.93 | 1.90 | 2.24 |  |  | 4.57 | 4.06 | 0.82 | 1.46 | 2.08 | 1.67 | 2.03 | 2.12 | 1.95 |
| Patron | 0.70 | 0.44 | 1.66 | 1.59 | 8.07 | 1.55 | 0.39 | 0.20 | 4.23 | 6.70 | 3.77 | 3.01 | 1.44 | 1.51 | 1.28 | 1.70 | 2.39 | 2.47 | 2.09 |
| Premium Crop | 1.55 | 2.16 | 2.77 | 0.91 | 4.05 | 3.84 | 0.51 | 0.24 | 9.43 | 11.01 | 4.42 | 4.67 | 2.47 | 2.75 | 3.38 | 4.65 | 3.68 | 3.35 | 3.78 |
| USVL 048 | 0.39 | 0.80 |  | 5.00 | 1.80 | 3.46 | 0.81 | 0.46 |  |  | 6.31 | 5.65 | 3.91 | 4.00 | 4.30 | 4.90 | 3.21 | 2.56 | 3.47 |
| USVL 093 | 0.31 | 0.69 | 2.11 | 2.59 | 2.86 | 2.59 | 0.66 | 0.75 |  |  | 5.99 | 5.87 | 3.99 | 4.37 | 4.61 | 6.35 | 3.13 | 2.61 | 3.32 |
|  |  |  |  |  |  |  |  |  |  |  |  |  |  |  |  |  |  |  |  |
|  |  |  |  |  |  |  |  |  |  |  |  |  |  |  |  | **Mean** | 2.60 | 2.37 | 2.63 |

**Table S6.** Neoglucobrassicin level (µ mol/g DW) of 23 cultivars grown under conventional (C) and organic (O) conditions in two locations (Maine and Oregon) in two seasons (Fall and Spring) from 2006-2008.

|  | **Maine** | | | | | | | | **Oregon** | | | | | | | | **Overall Cultivar Mean AHW** | **Overall Cultivar Mean C** | **Overall Cultivar Mean O** |
| --- | --- | --- | --- | --- | --- | --- | --- | --- | --- | --- | --- | --- | --- | --- | --- | --- | --- | --- | --- |
|  | **Fall** | | | | **Spring** | | | | **Fall** | | | | **Spring** | | | |  |  |  |
|  | **2006** | | **2007** | | **2007** | | **2008** | | **2006** | | **2007** | | **2007** | | **2008** | |  |  |  |
|  | **C** | **O** | **C** | **O** | **C** | **O** | **C** | **O** | **C** | **O** | **C** | **O** | **C** | **O** | **C** | **O** |  |  |  |
| Arcadia | 0.27 | 0.18 | 1.53 | 0.67 | 5.29 | 4.07 | 0.37 | 1.31 | 3.49 | 6.66 | 1.66 | 3.75 | 5.57 | 4.33 | 4.67 | 5.08 | 3.06 | 2.86 | 3.26 |
| B1 10 | 0.07 | 0.09 | 0.70 | 0.40 | 5.10 | 2.28 | 0.43 | 1.72 | 1.33 | 7.57 | 0.74 | 1.74 | 3.08 | 1.87 | 2.66 | 4.16 | 2.12 | 1.76 | 2.48 |
| Batavia |  |  | 0.97 | 0.74 | 4.96 | 2.98 | 1.07 | 0.90 |  |  | 4.12 | 4.39 | 6.01 | 3.91 | 3.09 | 8.66 | 3.48 | 3.37 | 3.60 |
| Beaumont |  |  |  | 1.38 | 3.87 | 3.05 | 0.70 | 0.64 |  |  | 1.10 | 1.45 | 6.17 | 9.16 | 7.63 | 7.30 | 3.86 | 3.89 | 3.83 |
| Belstar | 0.07 | 0.05 | 1.22 | 0.39 | 3.23 | 3.08 | 0.24 | 0.75 | 1.22 | 2.21 | 0.86 | 0.90 | 3.32 | 3.99 | 3.90 | 3.98 | 1.84 | 1.76 | 1.92 |
| Diplomat |  |  | 1.09 | 0.87 | 6.38 | 5.40 | 0.93 | 0.55 |  |  | 1.97 | 2.82 | 3.64 | 5.12 | 8.02 | 8.34 | 3.76 | 3.67 | 3.85 |
| Early Green | 0.32 | 0.31 | 0.73 | 0.39 | 2.87 | 1.83 | 2.03 | 1.03 | 2.63 | 3.66 | 1.90 | 3.45 | 4.97 | 1.53 | 1.87 | 9.00 | 2.41 | 2.17 | 2.65 |
| Everest | 0.18 | 0.16 | 0.48 | 0.68 | 2.97 | 3.25 |  |  | 4.25 | 4.29 | 1.30 | 3.77 | 3.22 | 3.26 | 3.94 | 6.83 | 2.76 | 2.33 | 3.18 |
| Fiesta | 0.10 | 0.16 |  | 0.86 | 3.61 | 4.02 | 1.29 | 1.14 | 1.97 |  | 1.43 | 1.90 | 7.32 | 4.54 | 8.53 | 7.92 | 3.20 | 3.46 | 2.93 |
| Green Goliath | 0.06 | 0.19 | 1.04 | 0.85 | 6.33 | 6.61 | 1.37 | 0.91 | 4.08 | 2.28 | 0.73 | 1.54 | 4.58 | 2.50 | 3.27 | 6.97 | 2.71 | 2.68 | 2.73 |
| Green Magic | 0.15 | 0.16 | 1.44 | 0.44 | 4.78 | 3.05 | 0.94 | 0.44 | 3.11 | 2.48 | 1.02 | 1.90 | 5.65 | 4.25 | 4.21 | 8.83 | 2.68 | 2.66 | 2.69 |
| Gypsy | 0.18 | 0.16 | 1.25 | 0.51 | 7.18 | 4.27 | 0.45 | 0.87 | 5.12 | 3.14 | 2.73 | 4.10 | 5.64 | 5.43 | 4.18 | 7.17 | 3.27 | 3.34 | 3.21 |
| Imperial |  |  |  | 1.08 |  | 3.86 | 0.40 | 0.66 |  |  | 1.22 | 1.65 | 4.40 | 4.61 | 4.48 | 6.63 | 2.90 | 2.63 | 3.08 |
| Marathon | 0.06 | 0.08 | 1.08 | 0.67 | 6.45 | 5.61 | 1.40 | 1.19 | 2.18 | 2.75 | 1.41 | 1.35 | 6.68 | 4.59 | 3.69 | 4.78 | 2.75 | 2.87 | 2.63 |
| Maximo | 0.11 | 0.15 | 1.13 | 1.22 | 7.37 | 4.67 | 0.45 | 0.38 | 5.15 | 8.32 | 2.21 | 2.92 | 5.05 | 4.68 | 2.86 | 5.12 | 3.24 | 3.04 | 3.43 |
| Nutribud | 0.13 | 0.10 | 0.71 | 0.10 | 1.95 | 1.54 | 0.94 | 0.34 | 3.31 | 1.39 | 0.73 | 1.06 | 1.03 | 0.77 | 0.99 | 2.31 | 1.09 | 1.22 | 0.95 |
| OSU OP | 0.28 | 0.29 | 0.43 | 0.24 | 2.86 | 4.60 | 1.34 | 0.51 | 3.79 | 3.06 | 1.63 | 2.51 | 2.74 | 2.52 | 1.95 | 5.62 | 2.15 | 1.88 | 2.42 |
| Packman | 0.10 | 0.04 | 0.14 | 0.12 | 0.99 | 0.33 | 0.28 | 0.33 | 1.04 | 1.40 | 0.35 | 0.91 | 0.88 | 1.67 | 0.51 | 1.81 | 0.68 | 0.54 | 0.83 |
| Patriot |  |  | 1.12 | 1.20 | 5.93 | 3.89 | 0.76 | 0.25 |  |  | 2.48 | 3.74 | 4.49 | 4.47 | 5.23 | 6.76 | 3.36 | 3.34 | 3.39 |
| Patron | 0.36 | 0.23 | 1.00 | 1.29 | 9.06 | 8.28 | 0.24 | 0.18 | 8.09 | 15.56 | 1.79 | 3.53 | 6.04 | 3.84 | 5.16 | 7.99 | 4.54 | 3.97 | 5.11 |
| Premium Crop | 0.44 | 0.49 | 1.32 | 0.15 | 6.27 | 3.68 | 0.32 | 0.73 | 3.39 | 8.12 | 2.12 | 2.63 | 4.99 | 6.08 | 2.21 | 4.71 | 2.98 | 2.63 | 3.32 |
| USVL 048 | 0.10 | 0.21 |  | 1.59 | 5.17 | 4.61 | 0.79 | 0.25 |  |  | 1.26 | 2.25 | 10.73 | 11.52 | 10.71 | 9.40 | 4.51 | 4.79 | 4.26 |
| USVL 093 | 0.14 | 0.53 | 1.08 | 1.23 | 8.33 | 4.73 | 0.35 | 0.58 |  |  | 1.48 | 1.54 | 6.23 | 8.22 | 6.99 | 6.95 | 3.46 | 3.51 | 3.40 |
|  |  |  |  |  |  |  |  |  |  |  |  |  |  |  |  |  |  |  |  |
|  |  |  |  |  |  |  |  |  |  |  |  |  |  |  |  | **Mean** | 2.90 | 2.80 | 3.01 |

**Table S7.** δ-tocopherol level (µ mol/g DW) of 23 cultivars grown under organic (O) and conventional (C) conditions in two locations (Maine and Oregon) in two seasons (Fall and Spring) from 2006-2008.

|  | **Maine** | | | | | | | | **Oregon** | | | | | | | | **Overall Cultivar Mean AHW** | **Overall Cultivar Mean C** | **Overall Cultivar Mean O** |
| --- | --- | --- | --- | --- | --- | --- | --- | --- | --- | --- | --- | --- | --- | --- | --- | --- | --- | --- | --- |
|  | **Fall** | | | | **Spring** | | | | **Fall** | | | | **Spring** | | | |  |  |  |
|  | **2006** | | **2007** | | **2007** | | **2008** | | **2006** | | **2007** | | **2007** | | **2008** | |  |  |  |
|  | **C** | **O** | **C** | **O** | **C** | **O** | **C** | **O** | **C** | **O** | **C** | **O** | **C** | **O** | **C** | **O** |  |  |  |
| Arcadia | 4.66 | 3.37 | 1.19 | 1.58 | 1.59 | 1.47 | 1.73 | 2.59 | 6.09 | 6.77 | 1.69 | 1.60 | 1.94 | 7.67 | 2.94 | 2.11 | 3.06 | 2.73 | 3.40 |
| B1 10 | 3.86 | 4.59 | 0.67 | 1.71 | 1.86 | 2.08 | 1.79 | 1.49 | 7.97 | 8.65 | 1.63 | 1.62 | 2.51 | 1.91 | 2.44 | 3.14 | 2.99 | 2.84 | 3.15 |
| Batavia |  |  | 0.73 | 0.84 | 1.69 | 0.95 | 1.33 | 1.13 |  |  | 0.98 | 0.98 | 2.17 | 1.92 | 0.58 | 2.14 | 1.29 | 1.25 | 1.33 |
| Beaumont |  |  |  | 0.57 | 1.34 | 1.33 | 1.74 | 1.32 |  |  | 0.82 | 2.12 | 1.89 | 1.75 | 1.35 | 2.95 | 1.56 | 1.43 | 1.67 |
| Belstar | 2.69 | 3.61 | 0.58 | 1.58 | 1.82 | 1.20 | 2.10 | 1.86 | 5.56 | 5.24 | 1.23 | 0.64 | 1.21 | 2.05 | 1.35 | 1.01 | 2.11 | 2.07 | 2.15 |
| Diplomat |  |  | 0.82 | 1.06 | 0.99 | 1.33 | 1.06 | 1.49 |  |  | 0.55 | 0.64 | 1.49 | 2.01 | 2.35 | 2.46 | 1.35 | 1.21 | 1.50 |
| Early Green | 6.91 | 8.43 | 1.07 | 1.97 | 3.12 | 3.12 | 1.43 | 1.44 | 7.54 | 8.14 | 0.74 | 0.81 | 1.53 | 1.66 | 2.38 | 1.95 | 3.26 | 3.09 | 3.44 |
| Everest | 9.08 | 10.38 | 1.38 | 1.20 | 1.25 | 2.85 |  |  | 7.87 | 8.70 | 2.12 | 2.90 | 1.30 | 2.29 | 2.02 | 3.38 | 4.05 | 3.57 | 4.53 |
| Fiesta | 3.42 | 2.26 |  | 0.78 | 1.16 | 1.27 | 1.95 | 0.47 | 8.08 |  | 1.93 | 2.58 | 3.22 | 1.26 | 1.64 | 2.65 | 2.33 | 3.06 | 1.61 |
| Green Goliath | 3.68 | 3.50 | 2.16 | 1.23 | 0.92 | 1.25 | 2.06 | 1.83 | 7.93 | 7.11 | 3.51 | 1.69 | 1.27 | 2.74 | 3.43 | 1.96 | 2.89 | 3.12 | 2.66 |
| Green Magic | 3.35 | 4.34 | 0.88 | 1.44 | 1.45 | 1.32 | 1.58 | 1.39 | 7.62 | 6.98 | 1.25 | 1.66 | 0.93 | 1.67 | 1.65 | 1.41 | 2.43 | 2.34 | 2.53 |
| Gypsy | 7.06 | 6.41 | 0.79 | 1.45 | 1.30 | 0.93 | 2.05 | 1.69 | 7.99 | 6.80 | 2.70 | 1.90 | 1.55 | 4.15 | 1.48 | 2.85 | 3.19 | 3.11 | 3.27 |
| Imperial |  |  |  | 2.18 |  | 1.95 | 1.37 | 1.07 |  |  | 1.20 | 1.21 | 2.39 | 3.62 | 1.68 | 1.40 | 1.81 | 1.66 | 1.91 |
| Marathon | 2.49 | 3.02 | 0.36 | 1.61 | 1.83 | 1.88 | 2.27 | 1.63 | 7.01 | 6.96 | 1.86 | 1.09 | 1.39 | 1.43 | 1.25 | 1.28 | 2.34 | 2.31 | 2.36 |
| Maximo | 2.10 | 1.25 | 0.75 | 1.18 | 3.37 | 1.94 | 1.74 | 3.92 | 6.15 | 6.26 | 1.08 | 1.14 | 1.66 | 1.83 | 1.94 | 1.29 | 2.35 | 2.35 | 2.35 |
| Nutribud | 2.84 | 3.70 | 1.49 | 1.35 | 3.69 | 1.96 | 2.11 | 1.56 | 7.47 | 6.21 | 0.86 | 1.08 | 1.32 | 1.25 | 1.19 | 3.07 | 2.57 | 2.62 | 2.52 |
| OSU OP | 14.64 | 21.86 | 0.76 | 1.87 | 2.40 | 2.51 | 3.99 | 3.42 | 9.31 | 6.79 | 0.95 | 1.87 | 1.71 | 1.85 | 3.97 | 3.23 | 5.07 | 4.72 | 5.42 |
| Packman | 8.98 | 6.80 | 1.58 | 1.57 | 1.41 | 3.86 | 1.66 | 1.85 | 6.10 | 7.19 | 1.75 | 0.94 | 1.45 | 1.54 | 2.96 | 3.10 | 3.30 | 3.24 | 3.36 |
| Patriot |  |  | 1.75 | 1.51 | 4.36 | 2.65 | 2.06 | 1.19 |  |  | 0.77 | 1.42 | 2.29 | 2.11 | 0.83 | 1.79 | 1.89 | 2.01 | 1.78 |
| Patron | 4.72 | 3.83 | 0.47 | 1.08 | 0.98 | 1.28 | 1.67 | 0.71 | 5.84 | 7.33 | 0.70 | 1.20 | 3.19 | 4.38 | 1.22 | 0.94 | 2.47 | 2.35 | 2.59 |
| Premium Crop | 3.04 | 7.38 | 0.70 | 0.72 | 1.92 | 1.65 | 1.97 | 1.46 | 6.74 | 7.66 | 0.89 | 1.48 | 1.60 | 1.72 | 2.71 | 2.38 | 2.75 | 2.45 | 3.06 |
| USVL 048 | 2.52 | 2.44 |  | 1.16 | 2.90 | 1.76 | 1.83 | 1.87 |  |  | 1.34 | 0.64 | 4.04 | 3.52 | 2.71 | 1.78 | 2.19 | 2.56 | 1.88 |
| USVL 093 | 2.46 | 2.75 | 2.00 | 2.17 | 8.63 | 1.52 | 2.58 | 2.03 |  |  | 1.35 | 1.77 | 2.69 | 2.86 | 2.87 | 2.03 | 2.69 | 3.22 | 2.16 |
|  |  |  |  |  |  |  |  |  |  |  |  |  |  |  |  |  |  |  |  |
|  |  |  |  |  |  |  |  |  |  |  |  |  |  |  |  | **Mean** | 2.61 | 2.58 | 2.64 |

**Table S8.** γ-tocopherol level (µ mol/g DW) of 23 cultivars grown under conventional (C) and organic (O) conditions in two locations (Maine and Oregon) in two seasons (Fall and Spring) from 2006-2008.

|  | **Maine** | | | | | | | | **Oregon** | | | | | | | | **Overall Cultivar Mean AHW** | **Overall Cultivar Mean C** | **Overall Cultivar Mean O** |
| --- | --- | --- | --- | --- | --- | --- | --- | --- | --- | --- | --- | --- | --- | --- | --- | --- | --- | --- | --- |
|  | **Fall** | | | | **Spring** | | | | **Fall** | | | | **Spring** | | | |  |  |  |
|  | **2006** | | **2007** | | **2007** | | **2008** | | **2006** | | **2007** | | **2007** | | **2008** | |  |  |  |
|  | **C** | **O** | **C** | **O** | **C** | **O** | **C** | **O** | **C** | **O** | **C** | **O** | **C** | **O** | **C** | **O** |  |  |  |
| Arcadia | 5.78 | 5.59 | 10.05 | 5.60 | 2.85 | 3.67 | 2.45 | 8.03 | 14.53 | 14.54 | 12.05 | 10.17 | 2.33 | 11.90 | 3.40 | 2.26 | 7.20 | 6.68 | 7.72 |
| B1 10 | 6.30 | 8.81 | 8.72 | 5.22 | 3.63 | 3.73 | 1.38 | 1.60 | 10.65 | 6.29 | 8.34 | 10.27 | 7.96 | 3.55 | 4.54 | 4.52 | 5.97 | 6.44 | 5.50 |
| Batavia |  |  | 4.07 | 2.99 | 2.56 | 3.22 | 2.46 | 5.19 |  |  | 7.84 | 6.55 | 6.86 | 5.43 | 1.77 | 1.61 | 4.21 | 4.26 | 4.17 |
| Beaumont |  |  |  | 8.96 | 1.51 | 2.38 | 1.67 | 3.50 |  |  | 13.77 | 14.39 | 5.63 | 3.50 | 1.93 | 2.53 | 5.43 | 4.90 | 5.88 |
| Belstar | 3.20 | 3.54 | 8.90 | 7.37 | 2.08 | 3.43 | 3.13 | 1.65 | 12.55 | 13.63 | 13.77 | 11.68 | 3.67 | 3.52 | 2.36 | 2.10 | 6.04 | 6.21 | 5.87 |
| Diplomat |  |  | 3.40 | 2.33 | 2.83 | 2.29 | 2.00 | 1.69 |  |  | 2.40 | 2.42 | 2.36 | 2.52 | 1.81 | 1.22 | 2.27 | 2.47 | 2.08 |
| Early Green | 2.60 | 3.51 | 1.58 | 0.95 | 2.52 | 3.23 | 2.67 | 1.71 | 5.25 | 5.87 | 3.95 | 2.16 | 3.85 | 2.89 | 2.44 | 2.68 | 2.99 | 3.11 | 2.88 |
| Everest | 4.01 | 9.35 | 8.21 | 3.94 | 3.19 | 4.59 |  |  | 13.89 | 14.26 | 16.65 | 16.75 | 6.02 | 4.08 | 13.71 | 14.13 | 9.49 | 9.38 | 9.59 |
| Fiesta | 4.49 | 3.76 |  | 8.69 | 2.23 | 2.59 | 5.25 | 4.23 | 15.30 |  | 12.76 | 12.27 | 8.11 | 4.08 | 2.88 | 3.04 | 6.40 | 7.29 | 5.52 |
| Green Goliath | 2.75 | 3.59 | 4.41 | 2.68 | 1.41 | 2.18 | 3.41 | 5.62 | 5.25 | 6.81 | 5.72 | 3.80 | 2.70 | 2.71 | 1.89 | 1.26 | 3.51 | 3.44 | 3.58 |
| Green Magic | 6.49 | 7.00 | 6.88 | 4.22 | 3.17 | 4.70 | 2.24 | 5.68 | 12.84 | 12.21 | 8.67 | 8.50 | 2.94 | 3.65 | 3.84 | 3.63 | 6.04 | 5.89 | 6.20 |
| Gypsy | 3.76 | 4.87 | 4.53 | 1.77 | 2.49 | 2.68 | 1.97 | 1.55 | 6.95 | 10.18 | 7.96 | 3.39 | 2.73 | 3.73 | 1.75 | 1.70 | 3.88 | 4.02 | 3.73 |
| Imperial |  |  |  | 3.60 |  | 2.91 | 3.20 | 4.46 |  |  | 3.30 | 2.37 | 4.42 | 1.72 | 1.18 | 1.35 | 2.85 | 3.02 | 2.73 |
| Marathon | 4.68 | 4.67 | 14.09 | 13.40 | 2.68 | 3.63 | 1.12 | 1.92 | 13.56 | 13.81 | 13.65 | 12.07 | 2.95 | 2.69 | 2.24 | 2.15 | 6.83 | 6.87 | 6.79 |
| Maximo | 3.02 | 3.97 | 16.92 | 2.35 | 3.80 | 4.89 | 1.53 | 1.65 | 4.41 | 4.73 | 14.70 | 12.01 | 5.33 | 8.07 | 4.20 | 2.68 | 5.89 | 6.74 | 5.04 |
| Nutribud | 2.05 | 3.57 | 1.94 | 0.99 | 3.25 | 3.21 | 2.60 | 2.62 | 15.49 | 14.46 | 2.37 | 2.32 | 2.32 | 2.98 | 1.92 | 2.58 | 4.04 | 3.99 | 4.09 |
| OSU OP | 6.79 | 6.53 | 1.82 | 2.47 | 5.10 | 4.17 | 1.95 | 2.71 | 6.62 | 6.23 | 4.39 | 4.78 | 4.76 | 5.71 | 3.55 | 2.10 | 4.35 | 4.37 | 4.34 |
| Packman | 3.40 | 2.82 | 1.89 | 1.18 | 4.83 | 5.10 | 2.60 | 1.82 | 5.32 | 8.87 | 2.79 | 2.40 | 2.27 | 2.04 | 2.04 | 2.71 | 3.26 | 3.14 | 3.37 |
| Patriot |  |  | 6.55 | 3.91 | 1.91 | 3.53 | 1.97 | 1.23 |  |  | 5.65 | 7.12 | 4.41 | 3.40 | 1.18 | 1.94 | 3.57 | 3.61 | 3.52 |
| Patron | 4.50 | 5.46 | 6.43 | 3.46 | 3.08 | 1.96 | 1.70 | 1.07 | 5.60 | 6.34 | 6.12 | 4.66 | 3.29 | 5.27 | 1.24 | 1.00 | 3.82 | 3.99 | 3.65 |
| Premium Crop | 3.83 | 7.98 | 1.31 | 1.30 | 1.94 | 1.93 | 2.22 | 1.05 | 6.03 | 4.60 | 1.43 | 2.24 | 1.66 | 1.58 | 1.32 | 2.52 | 2.68 | 2.47 | 2.90 |
| USVL 048 | 5.97 | 6.31 |  | 5.40 | 3.79 | 5.70 | 1.71 | 2.82 |  |  | 15.21 | 17.64 | 9.33 | 6.49 | 4.04 | 5.78 | 6.94 | 6.67 | 7.16 |
| USVL 093 | 2.84 | 3.96 | 9.87 | 8.93 | 6.63 | 3.06 | 1.52 | 1.92 |  |  | 12.52 | 17.04 | 3.00 | 3.03 | 2.06 | 1.95 | 5.60 | 5.49 | 5.70 |
|  |  |  |  |  |  |  |  |  |  |  |  |  |  |  |  |  |  |  |  |
|  |  |  |  |  |  |  |  |  |  |  |  |  |  |  |  | **Mean** | 4.92 | 4.98 | 4.87 |

**Table S9.** α-tocopherol level (µ mol/g DW) of 23 cultivars grown under conventional (C) and organic (O) conditions in two locations (Maine and Oregon) in two seasons (Fall and Spring) from 2006-2008.

|  | **Maine** | | | | | | | | **Oregon** | | | | | | | | **Overall Cultivar Mean AHW** | **Overall Cultivar Mean C** | **Overall Cultivar Mean O** |
| --- | --- | --- | --- | --- | --- | --- | --- | --- | --- | --- | --- | --- | --- | --- | --- | --- | --- | --- | --- |
|  | **Fall** | | | | **Spring** | | | | **Fall** | | | | **Spring** | | | |  |  |  |
|  | **2006** | | **2007** | | **2007** | | **2008** | | **2006** | | **2007** | | **2007** | | **2008** | |  |  |  |
|  | **C** | **O** | **C** | **O** | **C** | **O** | **C** | **O** | **C** | **O** | **C** | **O** | **C** | **O** | **C** | **O** |  |  |  |
| Arcadia | 18.52 | 19.92 | 28.67 | 28.13 | 43.15 | 47.18 | 12.59 | 30.50 | 40.93 | 55.38 | 44.89 | 44.77 | 34.17 | 66.58 | 47.56 | 45.04 | 38.00 | 33.81 | 42.19 |
| B1 10 | 23.67 | 26.19 | 27.36 | 25.81 | 41.10 | 45.27 | 24.37 | 43.80 | 24.97 | 26.24 | 36.39 | 43.23 | 48.91 | 46.38 | 52.42 | 57.61 | 37.11 | 34.90 | 39.32 |
| Batavia |  |  | 19.65 | 27.47 | 46.74 | 50.09 | 25.87 | 54.55 |  |  | 33.54 | 45.69 | 45.65 | 53.48 | 30.93 | 43.18 | 39.74 | 33.73 | 45.74 |
| Beaumont |  |  |  | 28.42 | 35.67 | 39.85 | 29.98 | 34.53 |  |  | 53.97 | 68.30 | 50.45 | 38.24 | 31.64 | 46.12 | 41.56 | 40.34 | 42.58 |
| Belstar | 14.62 | 21.15 | 29.08 | 36.28 | 48.00 | 54.64 | 24.37 | 16.95 | 45.17 | 43.37 | 56.56 | 53.28 | 57.97 | 52.86 | 57.37 | 52.14 | 41.49 | 41.64 | 41.33 |
| Diplomat |  |  | 41.16 | 39.46 | 38.39 | 37.87 | 23.49 | 34.48 |  |  | 39.32 | 37.01 | 34.72 | 37.07 | 33.59 | 32.29 | 35.74 | 35.11 | 36.36 |
| Early Green | 24.48 | 15.78 | 30.61 | 21.60 | 37.11 | 37.56 | 24.85 | 15.30 | 20.82 | 26.32 | 40.70 | 30.57 | 41.46 | 36.52 | 35.83 | 36.69 | 29.76 | 31.98 | 27.54 |
| Everest | 26.94 | 30.43 | 29.22 | 13.72 | 39.32 | 77.94 |  |  | 21.30 | 25.16 | 33.13 | 40.31 | 34.59 | 21.35 | 33.61 | 41.11 | 33.44 | 31.16 | 35.72 |
| Fiesta | 16.47 | 18.40 |  | 56.59 | 55.60 | 48.71 | 34.03 | 49.81 | 89.67 |  | 58.23 | 47.37 | 42.82 | 54.48 | 38.82 | 30.01 | 45.79 | 47.95 | 43.62 |
| Green Goliath | 23.51 | 16.11 | 27.59 | 37.59 | 30.98 | 33.13 | 36.95 | 25.79 | 39.12 | 60.12 | 54.86 | 41.53 | 36.93 | 39.84 | 36.82 | 37.89 | 36.17 | 35.85 | 36.50 |
| Green Magic | 27.78 | 21.78 | 23.79 | 18.08 | 47.82 | 45.41 | 40.54 | 45.12 | 67.34 | 57.56 | 33.14 | 30.09 | 26.42 | 31.42 | 33.13 | 43.80 | 37.08 | 37.49 | 36.66 |
| Gypsy | 31.91 | 30.25 | 33.84 | 25.32 | 46.16 | 58.50 | 28.45 | 36.89 | 29.07 | 24.74 | 36.60 | 36.73 | 33.13 | 44.23 | 41.09 | 40.20 | 36.07 | 35.03 | 37.11 |
| Imperial |  |  |  | 58.29 |  | 44.18 | 56.93 | 43.53 |  |  | 53.28 | 40.87 | 42.16 | 32.59 | 21.47 | 21.62 | 41.49 | 43.46 | 40.18 |
| Marathon | 22.98 | 13.52 | 38.33 | 49.72 | 42.17 | 45.21 | 22.25 | 29.47 | 69.94 | 66.72 | 35.34 | 40.32 | 32.00 | 39.61 | 57.52 | 49.54 | 40.92 | 40.07 | 41.76 |
| Maximo | 10.92 | 11.94 | 32.54 | 39.54 | 66.55 | 47.05 | 15.34 | 27.80 | 46.30 | 27.18 | 37.68 | 28.83 | 39.80 | 48.12 | 44.86 | 35.62 | 35.01 | 36.75 | 33.26 |
| Nutribud | 22.49 | 38.06 | 42.63 | 24.61 | 59.01 | 47.49 | 28.24 | 26.71 | 93.31 | 98.27 | 34.03 | 38.92 | 33.74 | 44.61 | 39.24 | 53.00 | 45.27 | 44.09 | 46.46 |
| OSU OP | 35.23 | 26.34 | 32.10 | 36.92 | 57.25 | 66.58 | 50.10 | 33.70 | 66.48 | 44.81 | 42.01 | 54.53 | 48.22 | 54.54 | 45.35 | 34.13 | 45.52 | 47.09 | 43.94 |
| Packman | 25.77 | 19.16 | 25.85 | 22.34 | 60.87 | 70.22 | 36.81 | 23.02 | 19.54 | 27.27 | 42.79 | 31.85 | 37.82 | 37.13 | 38.64 | 50.80 | 35.62 | 36.01 | 35.22 |
| Patriot |  |  | 31.46 | 39.00 | 25.15 | 54.30 | 41.73 | 29.44 |  |  | 36.15 | 33.82 | 42.47 | 44.55 | 45.76 | 49.24 | 39.42 | 37.12 | 41.73 |
| Patron | 29.19 | 44.64 | 35.59 | 35.83 | 50.27 | 44.31 | 29.85 | 25.59 | 36.20 | 42.14 | 32.92 | 33.99 | 39.27 | 60.25 | 45.31 | 37.34 | 38.92 | 37.33 | 40.51 |
| Premium Crop | 17.26 | 31.04 | 22.40 | 22.24 | 36.48 | 38.58 | 31.55 | 23.73 | 42.71 | 30.12 | 29.90 | 39.09 | 31.73 | 41.07 | 36.12 | 39.20 | 32.08 | 31.02 | 33.13 |
| USVL 048 | 15.47 | 11.36 |  | 34.50 | 58.46 | 60.23 | 33.38 | 48.83 |  |  | 39.84 | 51.39 | 68.83 | 49.07 | 55.43 | 55.08 | 44.76 | 45.24 | 44.35 |
| USVL 093 | 23.15 | 20.77 | 47.91 | 57.67 | 83.61 | 42.22 | 33.09 | 25.87 |  |  | 46.59 | 52.54 | 45.96 | 28.70 | 33.60 | 31.30 | 40.93 | 44.84 | 37.01 |
|  |  |  |  |  |  |  |  |  |  |  |  |  |  |  |  |  |  |  |  |
|  |  |  |  |  |  |  |  |  |  |  |  |  |  |  |  | **Mean** | 38.78 | 38.35 | 39.23 |

**Table S10.** Lutein level (µ mol/g DW) of 23 cultivars grown under conventional (C) and organic (O) conditions in two locations (Maine and Oregon) in two seasons (Fall and Spring) from 2006-2008.

|  | **Maine** | | | | | | | | **Oregon** | | | | | | | | **Overall Cultivar Mean AHW** | **Overall Cultivar Mean C** | **Overall Cultivar Mean O** |
| --- | --- | --- | --- | --- | --- | --- | --- | --- | --- | --- | --- | --- | --- | --- | --- | --- | --- | --- | --- |
|  | **Fall** | | | | **Spring** | | | | **Fall** | | | | **Spring** | | | |  |  |  |
|  | **2006** | | **2007** | | **2007** | | **2008** | | **2006** | | **2007** | | **2007** | | **2008** | |  |  |  |
|  | **C** | **O** | **C** | **O** | **C** | **O** | **C** | **O** | **C** | **O** | **C** | **O** | **C** | **O** | **C** | **O** |  |  |  |
| Arcadia | 10.03 | 8.00 | 9.46 | 11.63 | 12.18 | 16.09 | 16.02 | 12.04 | 20.50 | 17.44 | 10.06 | 12.98 | 4.45 | 25.14 | 18.35 | 14.97 | 13.71 | 12.63 | 14.79 |
| B1 10 | 13.35 | 11.12 | 11.48 | 12.86 | 16.50 | 19.26 | 8.82 | 13.04 | 20.27 | 11.78 | 10.56 | 13.07 | 21.29 | 20.00 | 22.93 | 19.72 | 15.38 | 15.65 | 15.11 |
| Batavia |  |  | 7.07 | 9.17 | 14.68 | 18.31 | 9.84 | 12.64 |  |  | 6.65 | 10.92 | 12.72 | 14.63 | 12.23 | 14.65 | 11.96 | 10.53 | 13.39 |
| Beaumont |  |  |  | 5.71 | 7.82 | 5.55 | 5.07 | 8.23 |  |  | 9.80 | 12.83 | 6.67 | 6.01 | 22.26 | 11.97 | 9.27 | 10.33 | 8.38 |
| Belstar | 3.46 | 5.23 | 5.56 | 6.49 | 8.86 | 13.16 | 12.56 | 14.82 | 20.40 | 16.75 | 10.15 | 9.19 | 9.15 | 15.15 | 11.49 | 7.50 | 10.62 | 10.20 | 11.03 |
| Diplomat |  |  | 11.34 | 20.27 | 17.15 | 24.65 | 18.61 | 25.22 |  |  | 12.58 | 16.47 | 17.32 | 18.39 | 21.61 | 17.53 | 18.43 | 16.44 | 20.42 |
| Early Green | 15.72 | 25.27 | 19.66 | 13.87 | 21.52 | 20.06 | 5.77 | 13.59 | 29.16 | 32.53 | 21.97 | 18.99 | 22.77 | 26.39 | 34.98 | 35.67 | 22.37 | 21.44 | 23.30 |
| Everest | 15.25 | 22.95 | 13.70 | 8.59 | 12.37 | 18.59 |  |  | 19.10 | 14.81 | 10.30 | 11.72 | 10.04 | 9.76 | 14.52 | 17.30 | 14.21 | 13.61 | 14.82 |
| Fiesta | 5.54 | 4.50 |  | 12.50 | 11.17 | 13.61 | 13.42 | 15.67 | 15.64 |  | 10.98 | 13.59 | 14.56 | 14.23 | 12.74 | 13.90 | 12.29 | 12.00 | 12.57 |
| Green Goliath | 14.24 | 11.72 | 10.74 | 11.53 | 15.62 | 14.88 | 10.98 | 14.48 | 19.57 | 17.54 | 13.05 | 11.15 | 11.61 | 25.72 | 17.79 | 16.27 | 14.81 | 14.20 | 15.41 |
| Green Magic | 21.47 | 13.29 | 11.13 | 10.74 | 12.69 | 15.30 | 9.78 | 7.25 | 26.75 | 22.92 | 16.80 | 12.86 | 10.81 | 12.32 | 15.05 | 18.78 | 14.87 | 15.56 | 14.18 |
| Gypsy | 12.15 | 19.10 | 8.30 | 8.14 | 17.86 | 14.94 | 14.85 | 16.72 | 16.85 | 14.75 | 8.36 | 11.70 | 9.90 | 15.85 | 15.58 | 16.99 | 13.88 | 12.98 | 14.78 |
| Imperial |  |  |  | 16.57 |  | 24.93 | 17.05 | 18.95 |  |  | 12.99 | 12.51 | 24.30 | 25.12 | 14.14 | 9.70 | 17.63 | 17.12 | 17.96 |
| Marathon | 8.05 | 4.76 | 7.22 | 11.45 | 14.70 | 14.44 | 17.43 | 10.21 | 25.52 | 23.64 | 7.39 | 9.56 | 10.75 | 8.39 | 13.27 | 11.47 | 12.39 | 13.04 | 11.74 |
| Maximo | 4.90 | 4.17 | 6.57 | 8.58 | 21.38 | 15.22 | 4.60 | 6.72 | 23.13 | 17.01 | 7.91 | 6.91 | 8.38 | 8.82 | 18.90 | 11.95 | 10.95 | 11.97 | 9.93 |
| Nutribud | 21.48 | 21.78 | 14.34 | 12.70 | 22.15 | 17.45 | 17.70 | 7.21 | 15.76 | 17.07 | 10.45 | 14.98 | 22.60 | 29.03 | 25.82 | 31.12 | 18.85 | 18.79 | 18.92 |
| OSU OP | 26.90 | 24.28 | 19.71 | 26.38 | 18.60 | 26.96 | 22.09 | 20.61 | 35.10 | 34.87 | 19.93 | 22.05 | 28.34 | 27.99 | 31.92 | 28.90 | 25.91 | 25.32 | 26.51 |
| Packman | 22.20 | 18.72 | 18.10 | 14.26 | 18.47 | 20.78 | 17.23 | 16.18 | 21.54 | 24.71 | 12.26 | 16.32 | 28.17 | 28.71 | 29.08 | 39.61 | 21.65 | 20.88 | 22.41 |
| Patriot |  |  | 8.91 | 12.42 | 12.73 | 21.82 | 20.33 | 18.82 |  |  | 8.59 | 8.57 | 15.96 | 11.81 | 16.00 | 19.76 | 14.64 | 13.75 | 15.53 |
| Patron | 21.52 | 22.76 | 8.61 | 11.78 | 13.58 | 15.14 | 23.34 | 22.18 | 18.17 | 14.78 | 9.27 | 8.76 | 11.30 | 26.18 | 21.12 | 15.93 | 16.53 | 15.86 | 17.19 |
| Premium Crop | 20.99 | 36.03 | 10.94 | 15.52 | 24.11 | 18.83 | 19.97 | 11.57 | 24.30 | 17.87 | 8.90 | 15.41 | 16.04 | 18.70 | 20.95 | 25.80 | 19.12 | 18.28 | 19.97 |
| USVL 048 | 3.60 | 2.34 |  | 19.49 | 26.00 | 11.36 | 20.14 | 29.02 |  |  | 5.95 | 6.67 | 7.41 | 14.91 | 7.81 | 5.71 | 12.34 | 11.82 | 12.78 |
| USVL 093 | 22.46 | 10.41 | 15.92 | 17.93 | 44.40 | 25.06 | 24.82 | 19.53 |  |  | 21.82 | 24.99 | 21.06 | 24.25 | 29.62 | 23.79 | 23.29 | 25.73 | 20.85 |
|  |  |  |  |  |  |  |  |  |  |  |  |  |  |  |  |  |  |  |  |
|  |  |  |  |  |  |  |  |  |  |  |  |  |  |  |  | **Mean** | 15.87 | 15.57 | 16.17 |

**Table S11.** Zeaxanthin level (µ mol/g DW) of 23 cultivars grown under conventional (C) and organic (O) and conditions in two locations (Maine and Oregon) in two seasons (Fall and Spring) from 2006-2008.

|  | **Maine** | | | | | | | | **Oregon** | | | | | | | | **Overall Cultivar Mean AHW** | **Overall Cultivar Mean C** | **Overall Cultivar Mean O** |
| --- | --- | --- | --- | --- | --- | --- | --- | --- | --- | --- | --- | --- | --- | --- | --- | --- | --- | --- | --- |
|  | **Fall** | | | | **Spring** | | | | **Fall** | | | | **Spring** | | | |  |  |  |
|  | **2006** | | **2007** | | **2007** | | **2008** | | **2006** | | **2007** | | **2007** | | **2008** | |  |  |  |
|  | **C** | **O** | **C** | **O** | **C** | **O** | **C** | **O** | **C** | **O** | **C** | **O** | **C** | **O** | **C** | **O** |  |  |  |
| Arcadia | 0.49 | 0.46 | 1.09 | 1.10 | 0.80 | 0.98 | 0.80 | 0.68 | 0.63 | 0.62 | 1.05 | 0.94 | 0.44 | 2.40 | 1.06 | 0.87 | 0.90 | 0.80 | 1.01 |
| B1 10 | 0.70 | 0.45 | 1.16 | 1.20 | 0.81 | 1.03 | 0.60 | 0.67 | 0.61 | 0.49 | 1.31 | 1.19 | 1.74 | 1.49 | 1.20 | 1.29 | 1.00 | 1.02 | 0.98 |
| Batavia |  |  | 0.73 | 0.93 | 1.71 | 0.92 | 0.67 | 0.81 |  |  | 0.63 | 1.01 | 1.06 | 1.15 | 0.76 | 0.89 | 0.94 | 0.93 | 0.95 |
| Beaumont |  |  |  | 0.67 | 0.97 | 0.50 | 0.64 | 0.52 |  |  | 1.00 | 1.13 | 0.80 | 0.62 | 1.42 | 0.81 | 0.83 | 0.97 | 0.71 |
| Belstar | 0.36 | 0.37 | 0.87 | 0.65 | 0.66 | 0.79 | 0.78 | 0.68 | 0.58 | 0.62 | 0.96 | 0.91 | 1.02 | 0.92 | 0.73 | 0.58 | 0.72 | 0.74 | 0.69 |
| Diplomat |  |  | 1.33 | 1.42 | 0.84 | 0.92 | 0.88 | 0.39 |  |  | 1.36 | 1.41 | 1.27 | 1.21 | 1.04 | 1.00 | 1.09 | 1.12 | 1.06 |
| Early Green | 0.82 | 0.96 | 1.82 | 1.22 | 1.02 | 1.18 | 0.51 | 0.55 | 0.74 | 0.76 | 1.94 | 1.26 | 1.32 | 1.22 | 2.19 | 1.93 | 1.22 | 1.30 | 1.13 |
| Everest | 0.66 | 0.97 | 1.22 | 0.95 | 0.73 | 1.36 |  |  | 0.58 | 0.53 | 0.79 | 0.87 | 0.41 | 0.47 | 0.87 | 1.01 | 0.81 | 0.75 | 0.88 |
| Fiesta | 0.38 | 0.39 |  | 1.14 | 0.73 | 0.83 | 0.82 | 0.78 | 0.61 |  | 0.81 | 0.86 | 1.01 | 1.18 | 0.73 | 0.72 | 0.78 | 0.73 | 0.84 |
| Green Goliath | 0.51 | 0.48 | 1.22 | 1.15 | 0.67 | 0.89 | 0.69 | 1.00 | 0.62 | 0.64 | 1.22 | 1.02 | 0.44 | 1.28 | 0.90 | 0.85 | 0.85 | 0.78 | 0.91 |
| Green Magic | 0.77 | 0.77 | 0.96 | 0.99 | 0.67 | 0.66 | 0.67 | 0.58 | 0.80 | 0.71 | 1.02 | 1.04 | 0.85 | 0.79 | 0.81 | 1.07 | 0.82 | 0.82 | 0.83 |
| Gypsy | 0.60 | 0.75 | 0.86 | 0.90 | 0.89 | 0.73 | 0.87 | 0.90 | 0.51 | 0.52 | 0.77 | 1.02 | 0.63 | 1.02 | 0.86 | 0.92 | 0.80 | 0.75 | 0.84 |
| Imperial |  |  |  | 1.52 |  | 1.15 | 0.76 | 0.82 |  |  | 1.18 | 1.11 | 2.06 | 0.96 | 1.21 | 0.73 | 1.15 | 1.30 | 1.05 |
| Marathon | 0.43 | 0.41 | 0.92 | 1.07 | 0.84 | 0.91 | 0.88 | 0.69 | 0.74 | 0.68 | 0.86 | 0.92 | 0.80 | 0.62 | 0.81 | 0.73 | 0.77 | 0.79 | 0.75 |
| Maximo | 0.37 | 0.35 | 0.95 | 1.01 | 1.49 | 1.05 | 0.51 | 0.57 | 0.66 | 0.57 | 0.84 | 0.71 | 0.75 | 0.72 | 0.93 | 0.68 | 0.76 | 0.81 | 0.71 |
| Nutribud | 0.57 | 0.59 | 1.34 | 1.24 | 1.09 | 0.86 | 0.96 | 0.59 | 0.58 | 0.54 | 1.03 | 1.45 | 1.08 | 1.24 | 1.24 | 1.37 | 0.99 | 0.99 | 0.98 |
| OSU OP | 0.88 | 1.68 | 1.62 | 1.81 | 0.97 | 1.98 | 1.62 | 1.08 | 0.91 | 0.75 | 1.60 | 1.78 | 1.54 | 1.75 | 1.33 | 1.30 | 1.41 | 1.31 | 1.51 |
| Packman | 0.84 | 0.78 | 1.67 | 1.31 | 0.89 | 1.51 | 0.62 | 0.99 | 0.62 | 0.68 | 1.06 | 1.41 | 1.37 | 1.13 | 1.37 | 1.69 | 1.12 | 1.05 | 1.19 |
| Patriot |  |  | 1.00 | 1.04 | 0.64 | 0.74 | 0.81 | 0.85 |  |  | 0.83 | 0.83 | 1.03 | 0.66 | 1.00 | 1.05 | 0.87 | 0.89 | 0.86 |
| Patron | 0.74 | 0.52 | 0.91 | 1.00 | 0.76 | 0.84 | 0.99 | 0.98 | 0.49 | 0.55 | 0.87 | 0.78 | 0.74 | 1.44 | 1.12 | 0.89 | 0.85 | 0.83 | 0.88 |
| Premium Crop | 0.62 | 0.83 | 1.08 | 0.97 | 0.97 | 0.97 | 0.91 | 0.57 | 0.72 | 0.63 | 0.88 | 1.41 | 0.88 | 0.85 | 1.00 | 1.27 | 0.91 | 0.88 | 0.94 |
| USVL 048 | 0.37 | 0.36 |  | 0.80 | 0.92 | 0.70 | 1.02 | 1.97 |  |  | 0.71 | 0.81 | 0.64 | 0.67 | 0.71 | 0.61 | 0.79 | 0.73 | 0.85 |
| USVL 093 | 0.49 | 0.50 | 1.48 | 1.50 | 1.73 | 1.00 | 1.44 | 1.06 |  |  | 1.66 | 1.78 | 1.24 | 1.12 | 1.66 | 1.39 | 1.29 | 1.39 | 1.19 |
|  |  |  |  |  |  |  |  |  |  |  |  |  |  |  |  |  |  |  |  |
|  |  |  |  |  |  |  |  |  |  |  |  |  |  |  |  | **Mean** | 0.94 | 0.94 | 0.95 |

**Table S12.** β-carotene level (µ mol/g DW) of 23 cultivars grown under conventional (C) and organic (O) conditions in two locations (Maine and Oregon) in two seasons (Fall and Spring) from 2006-2008.

|  | **Maine** | | | | | | | | **Oregon** | | | | | | | | **Overall Cultivar Mean AHW** | **Overall Cultivar Mean C** | **Overall Cultivar Mean O** |
| --- | --- | --- | --- | --- | --- | --- | --- | --- | --- | --- | --- | --- | --- | --- | --- | --- | --- | --- | --- |
|  | **Fall** | | | | **Spring** | | | | **Fall** | | | | **Spring** | | | |  |  |  |
|  | **2006** | | **2007** | | **2007** | | **2008** | | **2006** | | **2007** | | **2007** | | **2008** | |  |  |  |
|  | **C** | **O** | **C** | **O** | **C** | **O** | **C** | **O** | **C** | **O** | **C** | **O** | **C** | **O** | **C** | **O** |  |  |  |
| Arcadia | 6.12 | 4.40 | 13.21 | 15.60 | 25.55 | 31.85 | 12.12 | 40.12 | 29.87 | 28.17 | 22.92 | 28.97 | 17.41 | 23.15 | 20.82 | 15.37 | 20.98 | 18.50 | 23.45 |
| B1 10 | 15.90 | 14.77 | 17.19 | 22.07 | 34.26 | 44.78 | 9.87 | 28.06 | 28.80 | 18.29 | 28.87 | 33.54 | 34.00 | 32.47 | 26.75 | 37.30 | 26.68 | 24.46 | 28.91 |
| Batavia |  |  | 10.09 | 15.25 | 29.53 | 46.89 | 5.10 | 30.36 |  |  | 14.73 | 26.25 | 27.48 | 33.77 | 21.16 | 34.01 | 24.55 | 18.01 | 31.09 |
| Beaumont |  |  |  | 9.32 | 40.82 | 22.41 | 13.99 | 1.26 |  |  | 25.85 | 31.63 | 14.36 | 12.27 | 35.77 | 14.42 | 20.19 | 26.16 | 15.22 |
| Belstar | 1.25 | 2.15 | 9.25 | 11.88 | 17.93 | 24.37 | 17.70 | 17.84 | 36.24 | 37.90 | 26.73 | 16.10 | 13.81 | 15.24 | 18.57 | 12.26 | 17.45 | 17.68 | 17.22 |
| Diplomat |  |  | 30.24 | 30.96 | 43.26 | 38.36 | 21.01 | 26.11 |  |  | 26.92 | 31.53 | 29.41 | 25.00 | 28.68 | 23.98 | 29.62 | 29.92 | 29.32 |
| Early Green | 16.33 | 20.31 | 16.46 | 15.59 | 32.28 | 36.62 | 13.97 | 15.45 | 35.74 | 53.28 | 43.92 | 31.91 | 32.79 | 32.25 | 24.24 | 48.82 | 29.37 | 26.97 | 31.78 |
| Everest | 18.09 | 14.61 | 16.82 | 2.16 | 28.46 | 55.65 |  |  | 26.52 | 26.37 | 21.03 | 26.01 | 13.53 | 9.28 | 17.21 | 22.54 | 21.31 | 20.24 | 22.37 |
| Fiesta | 2.20 | 3.05 |  | 28.12 | 32.41 | 34.56 | 23.31 | 37.71 | 35.64 |  | 26.67 | 19.02 | 21.30 | 21.08 | 44.59 | 20.32 | 25.00 | 26.59 | 23.41 |
| Green Goliath | 25.33 | 15.65 | 14.94 | 23.06 | 23.12 | 36.39 | 22.40 | 12.98 | 35.45 | 38.49 | 29.97 | 23.78 | 18.37 | 24.57 | 36.16 | 36.36 | 26.06 | 25.72 | 26.41 |
| Green Magic | 23.60 | 14.28 | 17.77 | 9.13 | 31.83 | 13.52 | 23.28 | 22.59 | 51.25 | 42.92 | 22.12 | 20.98 | 15.91 | 28.43 | 32.16 | 28.17 | 24.87 | 27.24 | 22.50 |
| Gypsy | 12.83 | 11.77 | 12.39 | 6.69 | 43.92 | 35.55 | 28.99 | 43.78 | 22.45 | 23.06 | 13.54 | 16.64 | 15.90 | 23.89 | 29.42 | 24.34 | 22.82 | 22.43 | 23.21 |
| Imperial |  |  |  | 28.92 |  | 49.78 | 31.48 | 26.88 |  |  | 23.62 | 19.08 | 19.02 | 17.43 | 19.48 | 15.31 | 25.10 | 23.40 | 26.23 |
| Marathon | 5.01 | 2.17 | 16.76 | 23.46 | 28.80 | 36.01 | 25.07 | 25.58 | 53.44 | 48.81 | 13.15 | 24.44 | 16.38 | 25.14 | 29.05 | 28.40 | 25.10 | 23.46 | 26.75 |
| Maximo | 1.93 | 2.71 | 15.11 | 21.69 | 16.83 | 34.36 | 18.22 | 29.78 | 43.98 | 29.99 | 20.51 | 13.72 | 21.12 | 20.02 | 21.63 | 19.79 | 20.71 | 19.92 | 21.51 |
| Nutribud | 18.62 | 15.30 | 21.97 | 9.86 | 32.59 | 37.11 | 26.17 | 1.42 | 35.71 | 36.68 | 16.68 | 21.52 | 30.24 | 43.28 | 35.43 | 38.12 | 26.29 | 27.17 | 25.41 |
| OSU OP | 20.00 | 22.96 | 25.90 | 34.72 | 34.50 | 45.86 | 37.77 | 17.06 | 68.08 | 65.97 | 40.50 | 50.85 | 49.13 | 54.93 | 62.52 | 46.68 | 42.34 | 42.30 | 42.38 |
| Packman | 18.24 | 15.14 | 19.76 | 9.71 | 57.57 | 49.02 | 30.63 | 12.66 | 25.77 | 36.28 | 23.40 | 26.07 | 34.37 | 39.51 | 45.55 | 29.82 | 29.59 | 31.91 | 27.28 |
| Patriot |  |  | 15.23 | 18.26 | 15.57 | 58.73 | 38.21 | 33.25 |  |  | 16.21 | 16.67 | 23.32 | 22.89 | 10.94 | 17.69 | 23.91 | 19.91 | 27.91 |
| Patron | 19.09 | 20.31 | 15.60 | 18.19 | 41.37 | 31.79 | 33.11 | 30.70 | 34.24 | 31.97 | 19.03 | 14.57 | 20.22 | 35.81 | 21.13 | 19.23 | 25.40 | 25.47 | 25.32 |
| Premium Crop | 18.74 | 24.92 | 11.65 | 10.21 | 29.53 | 37.07 | 26.48 | 24.01 | 44.69 | 30.79 | 14.35 | 20.18 | 24.14 | 25.32 | 25.30 | 32.36 | 24.98 | 24.36 | 25.61 |
| USVL 048 | 0.98 | 0.36 |  | 23.70 | 44.16 | 25.88 | 28.52 | 34.06 |  |  | 13.50 | 14.81 | 15.43 | 27.79 | 20.38 | 9.68 | 19.94 | 20.49 | 19.47 |
| USVL 093 | 14.74 | 14.76 | 18.65 | 26.39 | 93.52 | 39.10 | 30.94 | 27.78 |  |  | 31.01 | 44.57 | 47.49 | 31.00 | 25.79 | 34.86 | 34.33 | 37.45 | 31.21 |
|  |  |  |  |  |  |  |  |  |  |  |  |  |  |  |  |  |  |  |  |
|  |  |  |  |  |  |  |  |  |  |  |  |  |  |  |  | **Mean** | 25.51 | 25.21 | 25.83 |
